# Recognition of Potential COVID-19 Drug Treatments through the Study of Existing Protein–Drug and Protein–Protein Structures: An Analysis of Kinetically Active Residues

**DOI:** 10.3390/biom10091346

**Published:** 2020-09-21

**Authors:** Ognjen Perišić

**Affiliations:** Big Blue Genomics, Vojvode Brane 32, 11000 Belgrade, Serbia; ognjen.perisic@gmail.com

**Keywords:** COVID-19, proteins, normal-modes, protein–drug interactions, chloroquine, ivermectin, remdesivir, sofosbuvir, boceprevir, α-difluoromethylornithine (DMFO), spike glycoprotein

## Abstract

We report the results of our in silico study of approved drugs as potential treatments for COVID-19. The study is based on the analysis of normal modes of proteins. The drugs studied include chloroquine, ivermectin, remdesivir, sofosbuvir, boceprevir, and α-difluoromethylornithine (DMFO). We applied the tools we developed and standard tools used in the structural biology community. Our results indicate that small molecules selectively bind to stable, kinetically active residues and residues adjoining them on the surface of proteins and inside protein pockets, and that some prefer hydrophobic sites over other active sites. Our approach is not restricted to viruses and can facilitate rational drug design, as well as improve our understanding of molecular interactions, in general.

## 1. Introduction

The *Coronaviridae* positive-stranded RNA virus family includes a substantial number of members, many of whom are known to cause a broad range of illnesses from the common cold to serious diseases like severe acute respiratory syndrome (SARS) and Middle East respiratory syndrome (MERS) [1,2]. The latest worldwide rapidly spreading disease Coronavirus disease 2019 (COVID-19) is caused by a new member of this virus family, Severe acute respiratory syndrome coronavirus 2 (SARS-CoV-2). The disease originally emerged in China in December 2019 with the most common symptoms being fever and cough, as well as shortness of breath, sore throat, headache, muscle ache, nausea, and diarrhea [1]. It can severely affect patients with immune systems weakened by pre-existing conditions, such as hypertension, diabetes mellitus, or cardiovascular diseases [3]. In some cases, symptoms also involve a still-unexplained loss of smell and taste [4]. It is assumed that the SARS-CoV-2 virus spreads through respiratory droplets, directly via physical contact, or through contact with contaminated objects. The virus spreads easier than SARS and MERS due to the high binding affinity between the virus spike glycoprotein (S) and the host receptor [5,6,7,8], making it more lethal. The virus directly affects the global economy by forcing countries to restrict access to work and slowing down supply lines, which causes a significant decline in gross national products worldwide, not encountered since the great depression.

There are a number of efforts and clinical trials underway to develop a vaccine and evaluate potential drugs for COVID-19, but such investigations usually take many months or even years to yield a successful treatment. Drug repurposing, on the other hand, may offer an immediate solution, because it considers already approved compounds as potential treatments for COVID-19.

There are, in general, two paths toward a viral treatment. One path directly attacks the virus and interrupts its replication machinery or its ability to attack host cells [9]. This path is often hard to implement due to the rapid emergence of new viral strains with acquired resistance to implemented drugs. The second path should therefore aim to block the host–viral interactions on the host side due to difficulties viral single point mutations should have in recovering the loss of host factors [10]. A recent study of human-virus protein–protein interactions (PPIs) detected 332 high-confidence SARS-CoV-2-human PPIs [11]. The study showed that 40% of SARS-CoV-2 proteins interact with endomembrane compartments or vesicle trafficking pathways and that viral proteins also interact with multiple innate immune pathways, the host translation machinery, bromodomain proteins, enzymes involved in ubiquitination regulation, and the cullin ubiquitin ligase complex. Importantly, it showed that the SARS-CoV-2 human PPI map is very similar to the interaction maps of West Nile virus (WNV) and Mycobacterium tuberculosis (Mtb). Among the human proteins involved in interactions with viral proteins, the study detected 66 druggable human (host) proteins targeted by 69 compounds (29 FDA-approved drugs, 12 drugs in clinical trials, and 28 preclinical compounds). It identified two groups of compounds with noticeable antiviral activity: inhibitors of mRNA translation/protein biogenesis (zotatifin, ternatin-4, PS3061, and plitidepsin), and predicted regulators of the sigma1 and sigma2 receptors (haloperidol, PB28, PD-144418, hydroxychloroquine, clemastine, cloperastine, progesterone, and the clinical molecule siramesine). The first group of compounds directly affects the viral cap-dependent mRNA translation because coronaviruses use the host translation machinery for their own mRNA translation. The compounds affecting the second group of proteins are approved and long-established human therapeutics [11]. As much as they are informative, such screening associative studies rarely offer detailed insights into mechanisms of molecular interactions. Structural studies [5,6,7,8,12], on the other hand, offer insights into residue and atom level physical contacts between molecules, but cannot offer general principles of molecular interactions.

In silico studies are widely used to screen potential drug candidates against COVID-19. Recently, Calligari et al. reported a molecular docking study of binding affinities between different viral proteins and several inhibitors, originally developed for other viral infections (hepatitis C virus (HCV), human immunodeficiency virus (HIV)) [13]. The examined drugs include simeprevir, saquinavir, indinavir, tipranavir, faldaprevir, ritonavir, lopinavir, asunaprevir, atazanavir, nelfinavir, amprenavir, darunavir, and fosamprenavir. Calligari et al. docked them with the 3C-like protease from SARS–CoV-2, the spike S SARS-CoV-2 protein, SARS-CoV-2 RNA-dependent RNA polymerase (RdRp), and nucleocapsid protein from SARS-CoV-2, and reported the corresponding binding free energies. The authors were able to select 5 of the 13 as potential inhibitors of SARS-CoV-2 protease. The three drugs tested with the spike S protein showed promise, and one presumably binds between the two monomers and interrupts transition between opened and closed states. A.A. Elfiky used molecular modeling, docking, and dynamics simulations to build a model for the viral RdRp protein and test its binding affinity to some clinically approved drugs and drug candidates [14]. The author concludes that sofosbuvir, ribavirin, galidesivir, remdesivir, favipiravir, cefuroxime, tenofovir, and hydroxychloroquine can tightly bind to the RdRp active site and can be good candidates for clinical trials. The author also noticed that the compounds setrobuvir, YAK, and IDX-184 can tightly wrap to the SARS-CoV-2 RdRp, and thus interrupt its function. That study also showed that the IDX-184-derived compounds (3,5-dihydroxyphenyl)oxidanyl and (3-hydroxyphenyl)oxidanyl can be effectively used to target SARS-CoV-2 RdRp. Yu et al. used AutoDock Vina software to screen potential drugs by estimating their binding free energies to the COVID-19 structural and non-structural protein sites [15]. They tested ribavirin, remdesivir, chloroquine, and luteolin, a compound present in honeysuckle. In traditional Chinese medicine, honeysuckle is generally believed to have antiviral effects. In this study, luteolin (the main flavonoid in honeysuckle) was found to bind with a high affinity to the same sites of the main protease of SARS-CoV-2 as the control molecule. De Oliveira et al. tested 9091 drug candidates by molecular docking against equilibrated SARS-CoV-2 spike S protein [16]. A total of 24 best-scored ligands, ivermectin among them, exhibited binding energies below −8.1 kcal/mol and were thus suggested as potential candidates. Interestingly, 14 of them are traditional herbal isolates and 10 are approved drugs. O. Santos-Filho used molecular docking to test HIV protease inhibitors against COVID-19 main protease [17]. The author showed that two non-natural compounds, danoprevir and lopinavir, and one herbal compound, corilagin, produced strong interactions with the inhibitor binding site of SARS-CoV-2 main protease. A modeling study by Pachetti et al. recognized a number of COVID-19 RdRp mutations that can affect drug treatments against COVID-19 [18]. B. R Beck et al. used a pre-trained deep neural network to identify commercially available drugs that could be used as treatments against SARS-CoV-2 [19]. They showed that atazanavir, an antiretroviral medication used to treat and prevent the human immunodeficiency virus (HIV), is the best compound against the SARS-CoV-2 3C-like proteinase, followed by remdesivir, efavirenz, ritonavir, dolutegravir, lopinavir, and darunavir. 

To facilitate the drug screening, we undertook a comparative in-silico study of binding modes in proteins targeted by six antiviral candidate drugs. We analyzed compounds that bind to parasitic and to human proteins. The drugs we have studied so far include (hydroxyl)chloroquine, ivermectin, remdesivir, sofosbuvir, boceprevir, and *α*-difluoromethylornithine (DMFO) (see Table 1). 

Chloroquine [20] and its less toxic derivative hydroxychloroquine [21] are drugs used to prevent and treat acute attacks of malaria. They are also used to treat discoid or systemic lupus erythematosus (SLE) and rheumatoid arthritis in patients whose symptoms have not improved with other treatments. These drugs are subject to several clinical trials worldwide as a potential treatment for COVID-19 [22,23]. Chloroquine has two potential modes of action against COVID-19, making it functional at both entry, and at post-entry stages of the COVID-19 infection [24]. It is known that it inhibits terminal glycosylation of the angiotensin-converting enzyme 2 receptor (ACE2) [25], that both SARS-CoV and SARS-CoV-2 use for cell entry [26,27]. ACE2 in non-glycosylated state may less strongly interact with the SARS-CoV-2 spike glycoprotein [26]. The second potential mode of action of chloroquine is based on the change of pH values in cell organelles, lysosome in particular [24,26]. Chloroquine passively diffuses through the cell in a lipophilic unprotonated state and in such state enters cell organelles [28]. Once it is inside the acidic environment of the lysosome, it becomes protonated and gets trapped within the vesicle. This raises the pH value and likely prohibits subsequent virus entry, fusion, or exit [26,28]. Interestingly, the above-mentioned study [11] indicates that PB28 is maybe ~20 times more potent viral inhibitor than hydroxychloroquine.

Ivermectin is a medication used to treat many various types of parasite infestations [29]. They include but are not limited to, head lice, scabies, river blindness (*onchocerciasis*), *strongyloidiasis*, *trichuriasis*, *ascariasis*, and *lymphatic filariasis*. Depending on the kind of treatment, the drug is taken by mouth or applied to the skin for external infestations. The molecular structure of ivermectin is rather complex and made of a set of macrocyclic lactone isomers. It binds to glutamate-gated chloride channels and increases the permeability of chloride ions [30]. Ion channels are attractive antiviral targets for two reasons. First, viruses can also have ion channels, active in viral assembly, morphogenesis, and viral release (e.g., E-protein in SARS-CoV [31]). Additionally, the inhibition of host (human) ion channels can be detrimental to viral replication, hepatitis C virus (HCV) in particular [32]. Ivermectin itself was shown to be able to inhibit the replication of SARS-CoV-2 in vitro [33]. All this indicates that ivermectin, as a modulator of chloride channel permeability, is a potential anti-viral drug. It is, therefore, currently the subject of clinical trials as a potential COVID-19 treatment [34]. 

Remdesivir is a nucleoside analog RdRp inhibitor initially developed to treat Ebola and Marburg virus diseases [9,35]. The drug decreases the viral RNA production by affecting the function of RdRp and proofreading by viral exoribonuclease (ExoN). Remdesivir is a subject of clinical trials as a potential COVID-19 treatment [36], as it was shown to reduce the lung viral load and improve pulmonary function with SARS infection [9]. 

Sofosbuvir is a medication used to treat HCV mono-infection and HCV/HIV-1 coinfection as a component of a combination antiviral regimen [37]. Sofosbuvir is a nucleotide prodrug that metabolically gets modified to the active uridine analog triphosphate, an inhibitor of HCV NS5B RNA-dependent polymerase. The inhibition of HCV NS5B RNA-dependent polymerase in turn suppresses viral replication. A. Sadeghi presented tentative results on the effectiveness of sofosbuvir and daclatasvir against COVID-19 [38]. 

Boceprevir is a medication used to treat chronic hepatitis C in untreated people or who do not react to ribavirin and peginterferon alfa alone [39]. It is used in combination with ribavirin (Copegus, Rebetol) and peginterferon alfa (Pegasys). It was shown to inhibit the COVID-19 (SARS-CoV-2) replication by inhibiting the virus’s main protease [40]. 

α-difluoromethylornithine **(DMFO)** (eflornithine), is a medication primarily used to treat *African trypanosomiasis* (sleeping sickness) and excessive facial hair in women [41]. Specifically, it is used for the second stage of sleeping sickness caused by *Trypanosoma brucei gambiense* and may be used in combination with nifurtimox [42]. It is used by injection or applied to the skin. The drug prevents the binding of the natural product ornithine to the active site of ornithine decarboxylase. We did not find any record of this drug ever being tried, to date, for COVID-19. However, since it is a halogenated organic molecule with somehow similar active sites as chloroquine, we decided to study it towards the treatment of COVID-19.

Here we report our research findings based on the method which we implemented to recognize protein–protein binding patterns, the self-adjustable Gaussian network model (SAGNM) [43]. The method predicts binding areas without any information on the binding partner’s properties, position, or orientation.

The SAGNM method is based on the Gaussian network model (GNM) formalism [44,45,46,47,48,49]. The GNM produces a set of vibrational modes via the eigenvalues and eigenvectors of the protein Kirchhoff contact matrix **Γ**. The fastest modes (with largest eigenvalues *λ*) are more localized and have steeper energy walls with a larger decrease in entropy and they are, therefore, referred to as kinetically hot residues [43].

The connection between kinetically hot residues and interfacial residues involved in protein–protein interactions has already been established [50]. The methodology introduced in [43], and used here, is based on a self-adjusting approach. It can effectively determine binding pockets and areas for peptides and small, drug-like molecules. It can pinpoint a segment on the surface of the protein where small ligands should bind. In most cases that area is one-tenth of the whole accessible surface area in the protein. 

The term “kinetically hot residues” is linguistically close, but does not carry the same meaning as the term “hot spots” that is often used in protein science. Hot spots are residues that often appear in structurally preserved interfaces (in more than 50% of cases). They are important because they are general contributors to the binding free energy. They are defined as spots where alanine mutation increases the binding free energy at least 2.0 kcal/mol [51,52,53,54,55,56,57].

## 2. Methods and Materials

To predict binding residues in proteins, we applied our self-adjustable interpretation of the Gaussian network model (SAGNM) [43]. The structure alignment, hydrophobicity calculation, visualization and analyses were performed with the programs Chimera [58] and Visual Molecular Dynamics (VMD) [59]. 

The self-adjustable Gaussian network model software is composed of several different programs. The first program calculates contact maps and the corresponding eigenvectors and eigenvalues [60] for each protein chain that forms a protein complex (given as a PDB file). Therefore, the code predicts binding residues without any knowledge of the position or orientation of the potential binding partners. In this case, the algorithm only analyzes the protein and does not analyze, nor use in any way, small ligands bound to it. The approach is based on the assumption that a protein preserves its conformation upon ligand attachment, which is often the case if the binding spot on the protein’s surface is hydrophobic [61]. 

The software first calculates the Kirchhoff contact matrix **Γ** for each protein. The matrix **Γ** calculation is based on the distances between Cα atoms only, and those distances have to be lesser or equal to 7 Å to consider two residues to be in contact [44,45,46,47]. The code then calculates and sorts **Γ** matrix eigenvalues and eigenvectors. The eigenvectors are sorted according to their corresponding eigenvalues. Those eigenvalues and eigenvectors are used in the second part that (iteratively) calculates the weighted sum of modes [47] as

(1) $${\langle {\left(\Delta R_{i}\right)}^{2}\rangle }_{k_{1}-k_{2}}=\left(3k_{B}T/\gamma \right)\sum_{k_{1}}^{k_{2}}\lambda_{k}^{-1}{\left[u_{k}\right]}_{i}^{2}/\sum_{k_{1}}^{k_{2}}\lambda_{k}^{-1}$$

where *λ_k_* are eigenvalues and **u***_k_* are eigenvectors. See the Supplementary Materials in [43] and references therein for details on the phantom network theory and GNM.

The product of this equation divided by $\left(3k_{B}T/\gamma \right)$ and normalized to produce mean square fluctuations of each residue for a given set of modes (*k*_1_ to *k*_2_). The equation produces an estimate of a kinetic contribution of each residue for that set of modes. The above equation is very similar to the singular value decomposition method [62] used in the linear least-squares optimization method. An additional code extracts contact and first layer residues. Finally, the third set of routines extracts neighboring residues and their distances for each residue per protein chain. That information is later used in the spatial spreading of the influence of kinetically hot residues.

The first step in the SAGNM analysis is the calculation of the weighted sum (Equation (1)). The procedure starts with a number of modes that correspond to the top 10% of the eigenvalues range of the analyzed protein. With normalized sum, only residues with an amplitude higher than 0.05 are perceived as hot residues. The number of hot residues is usually smaller than the number of potential contact or first layer residues (not contact residues with a spatial atom-atom distance of less than 4.5 Å from contact residues) to account for the fact that the influence of hot residues is spread to their sequential neighbors using the sequence information obtained from protein structure PDB files (to account for possible missing residues). The influence of hot residues is first spread to sequential neighbors only because proteins are polymer chains with physically connected residues. That implies that sequentially neighboring residues should exhibit correlated behavior. For chains longer than 100 amino acids (aa), hot residues, and eight their sequential neighbors upstream and downstream are labeled as predictions (four upstream, four downstream). For shorter chains, the influence is spread to six neighboring residues.

The prediction is then expanded to spatial neighbors. This approach is much closer to the true nature of the GNM algorithm that uses only spatial distances between Cα atoms and disregards any sequential/connectivity information. To apply this approach, the maximum cutoff Cα–Cα distance from the center of a hot residue was introduced to which its influence can be spread. The cutoff distance of 6 Å is applied with shorter protein chains (for sequence lengths shorter than 250 aa) and the cutoff of 8 Å with longer protein chains. All residues with Cα atoms within the sphere centered at the Cα atom of the hot residue and within the assigned cutoff distance are considered to be predictions. The two cutoff values were estimated empirically [43]. To extract spatial neighbors, distances between residues (Cα–Cα distances) were calculated for each particular protein and sorted in ascending order.

The self-adjustment GNM scheme is performed as follows:

**Step 1:**  Calculate the number of fast modes that correspond to the top 10% of the eigenvalues range.
**Step 2:**  Calculate the weighted sum (Equation (1)) and spread the influence of hot residues to sequential and spatial neighbors.
**Step 3a:**  If the overall percent of predictions is larger than a previously set value (for example, if the percent of predictions is larger than 30% of the total number of residues), the SAGNM procedure reduces the number of fast modes by one and goes to **Step 2**.
**Step 3b:**  If the percent of predictions is too small (e.g., less than 15% of all residues), the SAGNM procedure increases the number of fast modes by one and goes to **Step 2**.

The self-adjustable procedure repeats **Steps 2** and **3** until the percent of predictions fits between the maximum and minimum expected percentages for a given chain. To avoid infinite loops, only one increase followed by a decrease is allowed, and vice versa. Multiple consecutive increases or decreases are allowed. This approach ensures that longer proteins have enough predictions and that shorter ones are not saturated with too many false positives.

We focused our study on pdb structures with the listed drugs present as ligands. For chloroquine, we analyzed two structures, malarial parasite *Plasmodium Falciparum* lactate dehydrogenase (pdb id 1cet [63]) and human lysosomal protein saposin B (pdb id 4v2o [64]). The presence of saposin B in human lysosome makes it a logical target to analyze considering experimental evidence that the presence of chloroquine in lysosome inhibits coronavirus progression [24,26,28], and increased levels of lactate dehydrogenase have been shown to predict COVID-19 severity and mortality [65]. For ivermectin, we analyzed the binding pattern of the drug to the human glycine receptor alpha-3 (the glutamate-gated chloride channels (GluCls), pdb id 5vdh [66]) and *C. elegans* glycine receptor (pdb id 3rif [30]). For remdesivir, we analyzed its binding patterns in the recently released structure (pdb id 7bv2 [12]). We also performed the analysis of the binding pattern of the drug sofosbuvir to the hepatitis C virus (HCV) RdRp (pdb id 4wtg [67]) and compared them to the COVID-19 RdRp predictions (pdb id 6m71 [68]). Sofosbuvir was already analyzed in light of similarities between HCV and SARS-CoV-2 RdRp and similarities between remdesivir and sofosbuvir [68]. For boceprevir, we analyzed the structure SARS-Cov-2 main protease bound to the drug (pdb id 6wnp). For α-difluoromethylornithine, we analyzed a structure of *Trypanosoma brucei* ornithine decarboxylase (ODC) with D-ornithine bound to it (pdb id 1njj [69]). α-difluoromethylornithine binds to the active site of ODC and inhibits ornithine binding to it. We performed the comparative analysis of the binding patterns between the ACE2 human receptor and the spike glycoproteins from SARS (pdb id 6cs2 [70]) and SARS-CoV-2 (pdb id 6m0j [7]). We also analyzed the binding patterns between the SARS RBD with S230 human neutralizing antibody, and between SARS receptor-binding domain (RBD) and glycan shield (pdb id 6nb6 [71]).

## 3. Results

### 3.1. Chloroquine

**Figure 1:**
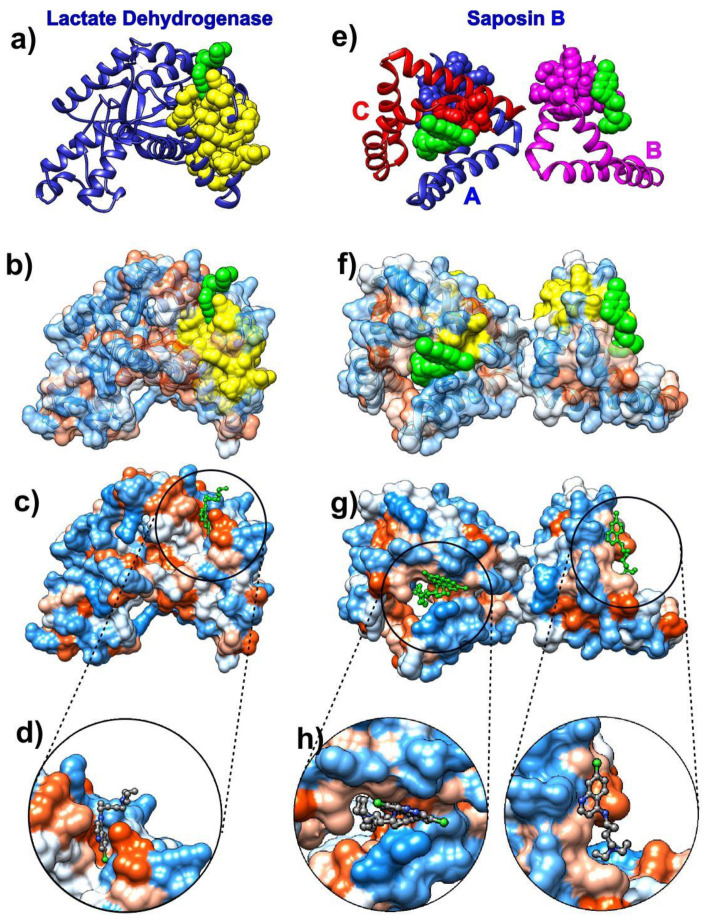
Chloroquine and its target proteins. Images on the left depict chloroquine bound to the cofactor binding site of *Plasmodium falciparum* lactate dehydrogenase (pdb id 1cet). Images on the right depict chloroquine bound to saposin B (pdb id 4v2o). (**a**) Lactate dehydrogenase is depicted as a blue ribbon, self-adjustable Gaussian network model (SAGNM) predictions are yellow and chloroquine green atoms. (**b**) Lactate dehydrogenase is depicted as a transparent hydrophobic surface (chain is visible as ribbon inside surface). SAGNM predictions are depicted as yellow atoms and chloroquine as green atoms. (**c**) Lactate dehydrogenase is depicted as an opaque hydrophobic surface and chloroquine as green balls and sticks. (**d**) The inset shows chloroquine within the hydrophobic pocket. (**e**) Saposin B chains are depicted as blue (chain A), pink (chain B), and red (chain C) ribbons. SAGNM predictions are depicted as blue, pink, and red atoms. Chloroquine molecules are shown as green atoms. (**f**) Saposin B is depicted as a transparent hydrophobic surface. SAGNM predictions are depicted as yellow atoms and chloroquine green atoms. (**g**) Saposin B is depicted as an opaque hydrophobic surface and chloroquine as green balls and sticks. (**h**) The two insets show chloroquine molecules within the hydrophobic pockets on the surface of the saposin B trimer. The figure is produced with the UCSF Chimera program [58].

The analysis of chloroquine binding patterns to *Plasmodium falciparum* lactate dehydrogenase (pdb id 1cet [63]) and human lysosomal protein saposin B (pdb id 4v2o [64]) reveals that chloroquine binds to kinetically active sites recognized by the SAGNM algorithm which are mostly hydrophobic (Figure 1).

Chloroquine binds selectively and competitively to the nicotinamide adenine dinucleotide (NADH) binding pocket of the lactate dehydrogenase enzyme and occupies a position similar to that of the adenyl ring of the cofactor. It is thus a competitive inhibitor for this critical glycolytic enzyme of malaria [63]. The SAGNM algorithm recognizes residues Val-24, Leu-25, Val-48, Leu-51, Ala-63, and Val-94 as hot. Their influence is spread to the residues Lys-20, Ala-21, Lys-22, Ile-23, Val-24, Leu-25, Val-26, Gly-27, Ser-28, Gly-29, Gly-32, Ala-37, Ile-40, Asn-44, Leu-45, Gly-46, Asp-47, Val-48, Val-49, Leu-51, Phe-52, Asp-53, Ile-54, Val-55, Pro-59, His-60, Gly-61, Lys-62, Ala-63, Leu-64, Asp-65, Thr-66, Ser-67, Cys-76, Lys-77, Val-78, Ser-79, Gly-80, Ser-81, Asp-87, Leu-88, Gly-90, Ser-91, Asp-92, Val-93, Val-94, Ile-95, Val-96, Thr-97, Ala-98, Ala-133, Phe-134, Ile-135, and Ile-136. See Figure 1a–d and Figure S1a in the Supplementary Materials. The drug interacts with residues Val-26, Gly-27, Phe-52, Asp-53, Ile-54, Tyr-85, Ala-98, Phe-100, Ile-119, and Glu-122, and the SAGNM algorithm correctly recognized residues 26, 27, 52, 53, 54, and 98. Other sites although exposed to solvent are not binding targets. The chloroquine molecule binds preferentially to hydrophobic sites (see Figure 1c,d) and avoids neutral and hydrophilic areas.

The lysosomal protein saposin B is a trimer formed by chains A, B, and C. It selectively degrades lipids and is one of the most studied members of the saposin protein family [64]. Its deficiency or malfunctioning leads to the accumulation of lipids in the lysosome and results in the lysosomal storage disease metachromatic leukodystrophy (see [64] and references therein). The SAGNM algorithm recognizes the residues Ile-8 and Cys-71 in chain A, Ile-8, and Cys-71 in chain B and Cys-71 in chain C as hot. Their influence is spread to the residues Gln-5, Asp-6, Cys-7, Ile-8, Gln-9, Met-10, Val-11, Pro-67, Lys-68, Glu-69, Ile-70, Cys-71, Ala-72, Leu-73, Val-74, Phe-76, and Cys-77 in chain A; to the residues Gln-5, Asp-6, Cys-7, Ile-8, Gln-9, Met-10, Val-11, Pro-67, Lys-68, Glu-69, Ile-70, Cys-71, Ala-72, Leu-73, Val-74, Phe-76 and Cys-77 in chain B; and to the residues Lys-68, Glu-69, Ile-70, Cys-71, Ala-72, Leu-73, Val-74, Phe-76 and Cys-77 in chain C. See Figure 1e–h and Figure S1b in the Supplementary Materials. The SAGNM recognizes that the chloroquine molecules interact with residues Glu-69 and Leu-73 from chain B, out of residues Ala-58, Met-61, Met-65, Glu-69, and Leu-73. With chain C, it recognizes residues Glu-69 and Leu-73, out of residues Met-61, His-64, Met-65, Glu-69, and Leu-73. With chain A, it does not emphasize the residue Arg-38, but it recognizes the binding patch with the chain C (see Figure 1e).

With both chloroquine examples, the expected number of predictions for the SAGNM algorithm was set to be between 10% and 15%, and this corresponds to the fastest normal mode for each chain. Our results suggest that chloroquine’s binding to COVID-19 proteins should follow the same patterns as with lactate dehydrogenase and saposin B. Namely, it should attach to residues which are both hydrophobic and kinetically active (or very close to kinetically active sites).

The analysis of chloroquine’s nondiscriminatory binding to human and parasitic proteins may explain its efficiency against parasitic infections as well as offer a glimpse into its toxicity.

### 3.2. Ivermectin

The drug ivermectin binds glutamate-gated chloride channels and thus increases their permeability to chloride ions. We analyzed the ivermectin’s binding to the human glycine receptor alpha-3 (pdb id 5vdh [66]). This structure, besides ivermectin, also has glycine and the potentiator AM-3607 (7c6) bound to the glycine receptor. The comparison of the crystal structure used in this research to previously determined structures revealed that the ivermectin binding expands the ion channel pore [66].

**Figure 2:**
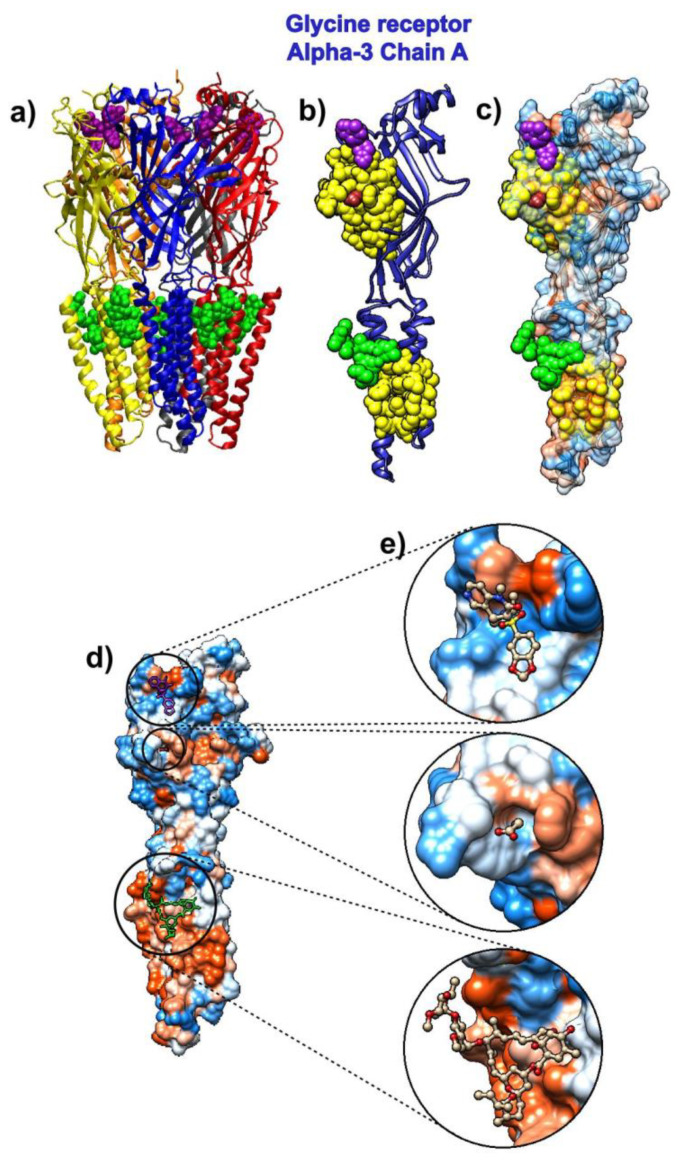
Ivermectin and its target protein, human glycine receptor alpha-3 (pdb id 5vdh). (**a**) Five pentamer chains (A to E) are represented as ribbons. Ivermectin is represented via green atoms. Glycine molecules are brown and represented as atoms, and 7C6 molecules are represented as purple atoms. (**b**) Chain A from human glycine receptor Alpha-3 is represented as a blue ribbon. SAGNM predictions are depicted as yellow atoms. Ivermectin is represented via green atoms. The glycine molecule is brown and represented as spherical atoms. The 7C6 molecule is represented as purple atoms. (**c**) Chain A from human glycine receptor alpha-3 is depicted as a transparent hydrophobic surface. SAGNM predictions are yellow, glycine molecule is represented as brown, 7C6 molecule as purple, and ivermectin as green atoms. (**d**) Chain A from human glycine receptor alpha-3 is depicted as an opaque hydrophobic surface. The glycine molecule is represented as brown balls and sticks, the 7C6 molecule is represented as purple, and ivermectin as green balls and sticks. (**e**) The three insets show glycine, 7C6, and ivermectin molecules inside the hydrophobic pockets on the surface of the chain A of human glycine receptor alpha-3. The figure is produced with the VMD and UCSF Chimera programs [58,59].

The receptor is a pentamer, so we only analyzed the binding to its chain A (Figure 2). The SAGNM algorithm recognized the residues Glu-157, Ser-158, Phe-168, Phe-207, Thr-208, Cys-209, Ile-210, Glu-211, Ser-238, Gly-256, Thr-259, Val-260, Val-294, and Leu-298 as kinetically hot. For the list of residues, their influence is spread to see the list below Figure S2 in the Supplementary Materials. The expected number of predictions for the SAGNM algorithm was set to be between 25% and 30%, and this corresponds to the three fastest modes. The larger size of alpha-3 chains required the increase in the expected number of predictions in comparison to the chloroquine. The three compounds bind to the residues Arg-27, Ile-28, Arg-29, Phe-32, Phe-159, Gly-160, Tyr-161, Asp-165, Tyr-202, Thr-204, Phe-207, Ser-267, Ser-268, Ser-278, Val-280, Asp-284, Ala-288, Leu-291, Leu-292 and Phe-295. The SAGNM algorithm recognized residues Phe-159, Gly-160, Tyr-161, Asp-165, Tyr-204, Phe-207, Leu-291, Leu-292 and Phe-295. The analysis (Figure 2) reveals that all three compounds (ivermectin, glycine, and AM-3607 (7c6)) bind to kinetically active and residues adjoining them [43], some of which are highly hydrophobic, with ivermectin binding almost exclusively hydrophobic residues. This means that this drug seeks similar sites on the surface of the COVID-19 proteins.

The SAGNM analysis of the *C. elegans* glycine receptor (pdb id 3rif, chain B) is given in the Supplementary Materials (Figure S3). As with human glycine receptor, the small ligands are bound to residues in chain B of 3rif protein complex recognized by the SAGNM algorithm, which indicates that the binding process follows a similar pattern in both proteins. A drug to bind the chloride channel should probably bind to such residues.

### 3.3. Remdesivir

**Figure 3:**
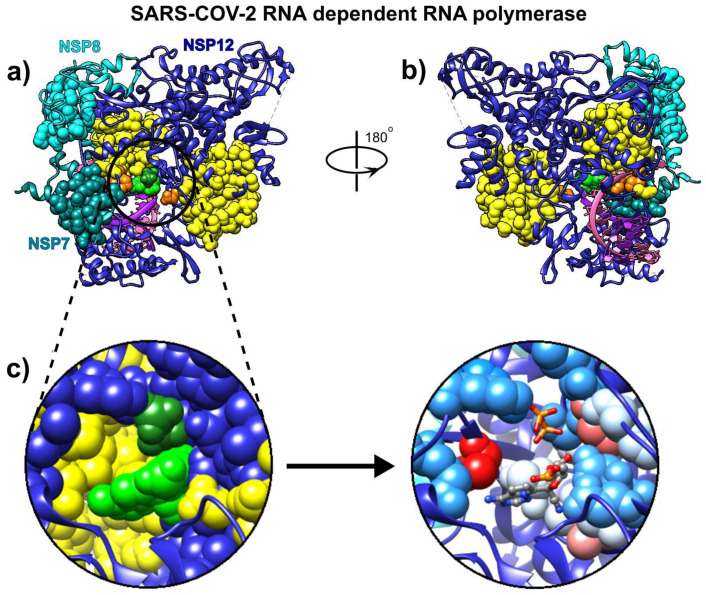
Remdesivir bound to the primer RNA inside the central channel of SARS-CoV-2 RNA-dependent RNA polymerase (RdRp), NSP12 (pdb id 7bv2 described in [12]). (**a**) Three RNA polymerase chains, NSP 12, NSP7, and NSP8, are represented as blue, cyan, and dark cyan ribbons. Remdesivir is represented as green atoms and pyrophosphate as dark green atoms. The dashed lines represent protein segments missing from the deposited structure. (**b**) The same structure rotated approximately 180° around the vertical axis. (**c**) Remdesivir and pyrophosphate inside the binding pocket, surrounded by the yellow SAGNM predictions (left), and inside the pocket with contact residues colored by hydrophobicity.

We used the recently cryo-EM determined structure of SARS-CoV-2 RdRp with double-stranded template-primer RNA and remdesivir (pdb id 7bv2 [12]) to analyze the RNA and drug binding to residues in RdRp. The structure reveals that the double-stranded RNA is inserted into RdRp’s central channel and that the active triphosphate form of remdesivir is covalently bound to the primer strand at the first replicated base, which effectively terminated the chain elongation (the prodrug form of remdesivir does not have any inhibitory effect on the polymerization activity of the purified enzyme [12]). The SAGNM algorithm recognized the residues Gly-503, Thr-538, Ile-539, Thr-540, Gln-541, Ala-558, Val-560, Ser-561, Val-609, His-613, Glu-665, Met-666, Val-667, Met-668, Ala-702, Ala-706, Phe-753, Cys-765, and Asn-767 in chain A as hot; the residues Asp-161 and Ile-185 in chain B as hot; and the residues Lys-7, Ser-10, His-36, Ile-39, Ala-48 and Lys-51 in chain C as hot—see Figure 3. For the predictions, see the list below Figure S4 in the Supplementary Materials.

Our analysis reveals that the residues recognized via the fastest two normal modes (for the expected number of predictions between 10% and 15%) delineate the central channel (Figure 3a,b). The enzymatically important residues Lys-500, Ser-501, Lys-545, and Arg-555 are all recognized by the SAGNM algorithm using just the fastest normal mode, while the residue Asp-761 of the catalytic center (out of residues Ser-759, Asp-760, and Asp-761 that form the catalytic center) is also emphasized with the two fastest modes. Residues Lys545 and Arg-555 are important because they stabilize the incoming nucleotide in the correct position for catalysis. The crystal structure shows that the catalytic center of RdRp, NSP12 protein (Non-Structural Protein 12), does not have any contacts with base pairs of RNA emphasizing RdRp’s sequence-agnostic polymerization ability [12]. This is in concordance with our coarse-grained analysis, based on the positions of C-α atoms only, that shows that stable, kinetically active residues outline the enzyme’s central channel.

### 3.4. Sofosbuvir

**Figure 4:**
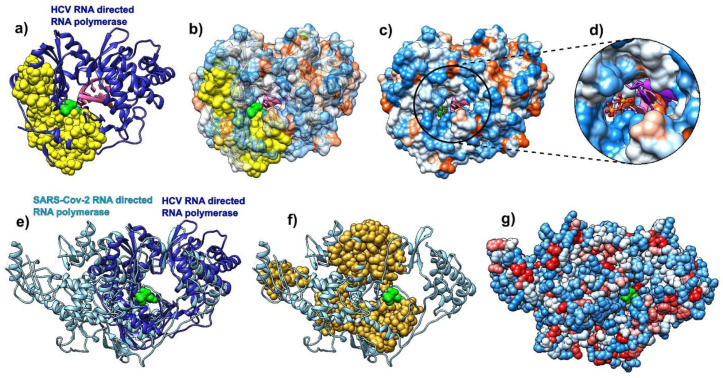
Comparative analysis of hepatitis C virus (HCV) (pdb id 4wtg, chain A, left) bound to sofosbuvir, and COVID-19 RNA directed RNA polymerase (RdRp, pdb id 6m71, chain A, right). (**a**) HCV RNA-directed RNA polymerase is depicted as a blue ribbon, RNA is purple, and sofosbuvir is a green molecule (full atom representation). (**b**) HCV RdRp is represented as a transparent hydrophobic surface, SAGNM predictions are yellow and sofosbuvir is represented via green atoms. (**c**) HCV RdRp is represented as an opaque hydrophobic surface, and sofosbuvir is represented via green sticks. (**d**) The inset shows sofosbuvir inside the polymerase catalytic core. (**e**) HCV RdRp (blue ribbon) structurally aligned with COVID-19 RdRp (light blue ribbon). Sofosbuvir is a green molecule inside the HCV RdRp catalytic core. (**f**) COVID-19 RdRp as a light blue ribbon. SAGNM predictions are dark yellow atoms. Sofosbuvir is a green molecule inside the catalytic core. The position stems from the structurally aligned HCV RdRp. (**g**) COVID-19 RdRp as hydrophobically colored atoms (residues hydrophobicities). Sofosbuvir is a green molecule inside the catalytic core. The position stems from the structurally aligned HCV RdRp. With COVID-19 RNA polymerase, sofosbuvir’s position corresponds to the position it has when bound to HCV RNA polymerase.

We performed a comparative analysis of the hepatitis C virus (HCV) RdRp (chain A in pdb id 4wtg [67]; the structure is given with the drug sofosbuvir bound to it) and the COVID-19 RdRp (chain A in pdb id 6m71 [68]). We followed the steps of Y. Gao and collaborators [68] and attempted to compare predictions of the binding residues in HCV RdRp to sofosbuvir, to binding residues predictions in SARS-CoV-2 RdRp. The binding residues in HCV are buried deep inside the polymerase catalytic core. Our analysis shows that they are generally delineated by the SAGNM predicted residues and are thus stable. They are recognized by the fastest two normal modes (for the expected number of predictions set to be between 15% and 20% of all residues) (Figure 4a), but they are not explicitly hydrophobic (Figure 4b,c). The SAGNM algorithm recognized the residues Met-139, Ala-157, Met-266, Asn-268, Cys-279, Lys-298, Phe-339, and Met-343 of the HCV RdRp (pdb id 4wtg) as hot. The algorithm recognized the residues Met-139, Ala-157, Met-266, Asn-268, Cys-279, Lys-298, Phe-339, and Met-343 of the main enzymatic unit of COVID-19 RdRp (pdb id 6m71) as hot. For the full list of hot residues and predictions for HCV RdRp, see Figure S5 in the Supplementary Materials and the list below it, and for COVID-19 RdRp, see Figure S6 and the list below it.

The structural alignment of HCV and COVID-19 RdRp (Figure 4e) using the Chimera program [58] shows that they share the structure of the binding pocket, and also reveals that the catalytic cores in both proteins are bounded by the SAGNM predictions, but the overall distribution of residues is only partially similar between the two proteins (Figure 4f). In both cases, the expected number of targets is between 15% and 20%. With HCV RdRp, this corresponds to the two fastest modes, and with COVID-19 RdRp and the main catalytic unit NSP12, it corresponds to the seven fastest modes. The similarities suggest that the interior of the RdRp in coronaviruses are attractive binding spots for small compounds in general.

**Figure 5:**
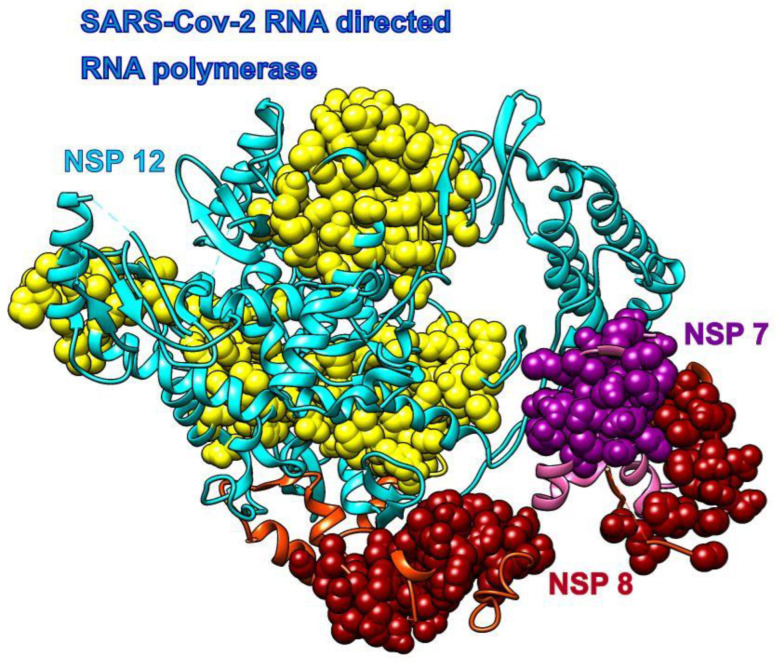
COVID-19 RNA directed RNA polymerase with cofactors NSP7 and NSP8 (pdb id 6m71). The NSP 12 chain is cyan, and its SAGNM predictions are yellow. The NSP 7 chain is pink and its SAGNM predictions are purple. The NSP 8 chain is orange and SAGNM predictions are dark red. The dashed lines represent segments missing from the coordinates file.

The main enzymatic unit of COVID-19 RdRp, NSP12, mostly keeps its conformation between RNA free and RNA bound structures [12]. Figure 5 shows that cofactors NSP7 and NSP8 seek patches with kinetically active residues on the surface of NSP12, but they are also in contact with kinetically less active areas. This should be analyzed in light of the fact that SARS-CoV-2 RdRp (NSP12) cannot perform its function without NSP7 and NSP8 [12]. The distribution of kinetically very active and kinetically dormant residues may be important for the overall stability of NSP12. Such distribution can also act as a stochastic oscillator/transformer that translates random fluctuations of solvent and proteins into regular vibrations that produce a regular rhythm of translation (i.e., act as a chemical clock/oscillator).

### 3.5. Boceprevir

**Figure 6:**
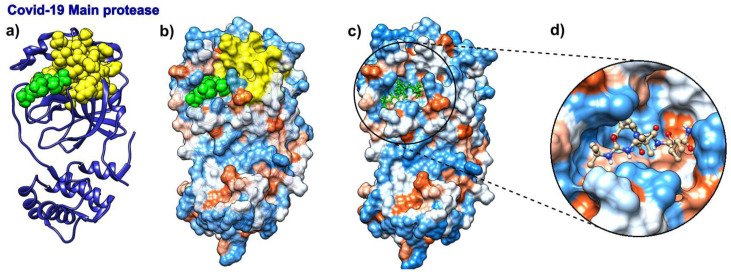
Boceprevir and its target protein COVID-19 (SARS-CoV-2) main protease (pdb id 6wnp). (**a**) COVID-19 Main protease is depicted as a blue ribbon, SAGNM predictions are yellow, and boceprevir as green atoms. (**b**) COVID-19 main protease is depicted as a transparent hydrophobic surface, SAGNM predictions are yellow, and chloroquine is a green molecule. (**c**) COVID-19 main protease is depicted as an opaque hydrophobic surface, and chloroquine is depicted via green balls and sticks. (**d**) The inset shows boceprevir inside the binding pocket.

We analyzed the binding modes in SARS-CoV-2 main protease with boceprevir bound to it (pdb id 6wnp). With the expected number of targets set between 15% and 20% of the total number of residues, which corresponds to the fastest normal mode, the SAGNM algorithm recognized the residues Val-20, Asn-28, and Cys-38 as kinetically hot in the SARS-Cov-2 main protease (pdb id 6wnp). This corresponds to the predictions of Cys-16, Met-17, Val-18, Gln-19, Val-20, Thr-21, Cys-22, Gly-23, Thr-24, Thr-25, Thr-26, Leu-27, Asn-28, Gly-29, Leu-30, Trp-31, Leu-32, Asp-34, Val-35, Val-36, Tyr-37, Cys-38, Pro-39, Arg-40, His-41, Val-42, Phe-66, Leu-67, Val-68, Gln-69, Val-86, Leu-87, Lys-88, Cys-117, Tyr-118, Asn-119, Gly-120, Gly-143, Ser-144, Cys-145, Gly-146, Ser-147 and Met-162. Of all the residues in contact with boceprevir, the SAGNM algorithm recognized the residues Thr-25, Thr-26, Leu-27, His-41, Gly-143, Ser-144, and Cys-145—see Figure 6. See also Figure S7 in the Supplementary Materials for the distribution of hot and predicted residues.

### 3.6. Eflornithine

**Figure 7:**
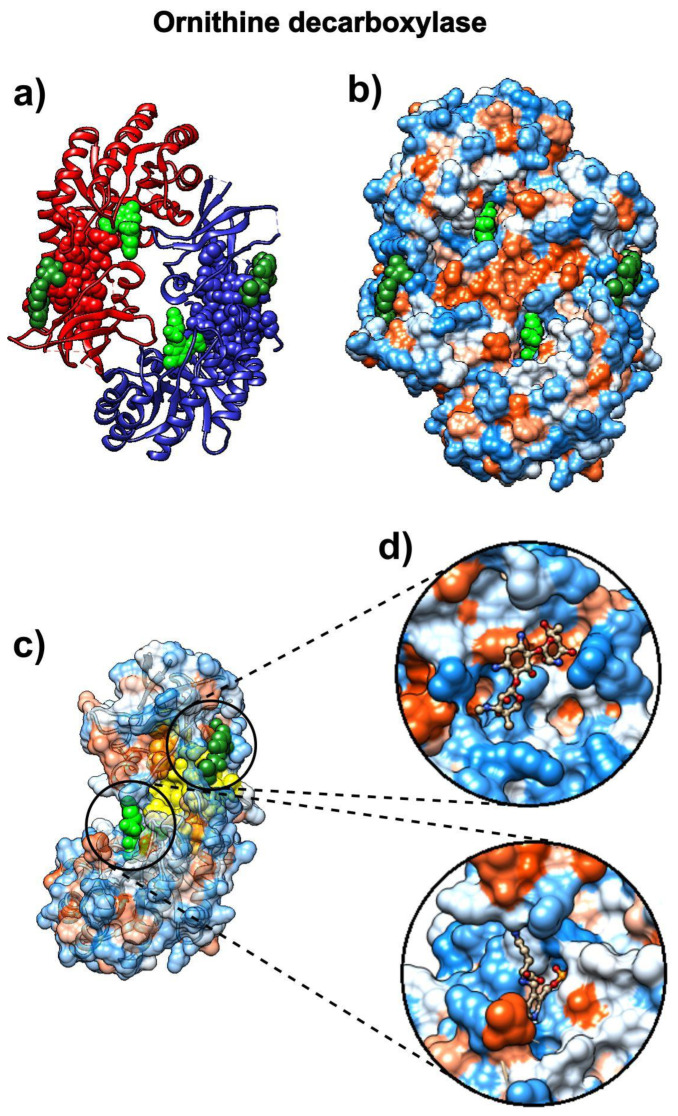
D-ornithine and its target protein *Trypanosoma brucei* ornithine decarboxylase (pdb id 1njj). (**a**) Ornithine decarboxylase chains A (red) and B (blue) are depicted as ribbons, with D-ornithine and G-418 as green and dark green molecules, respectively. (**b**) Ornithine decarboxylase chains A and B are depicted as hydrophobicity surface, with D-ornithine and G-418 as green and dark green molecules, respectively. (**c**) Ornithine decarboxylase chain A depicted as a transparent hydrophobicity surface, with SAGNM predictions are yellow atoms, and with D-ornithine and G-418 as green and dark green molecules. (**d**) D-ornithine and G-418 molecules depicted as colored bonds and sticks, correspondingly to the atom type and inside pockets on the surface of ornithine decarboxylase.

The drug α-difluoromethylornithine (DMFO, eflornithine) prohibits binding of the natural non-coded amino acid ornithine to the active site on the surface of *Trypanosoma brucei* ornithine decarboxylase (ODC, pdb id 1njj [69]). The binding of this drug should follow the binding patterns of ornithine. Figure 7 shows that the SAGNM algorithm accurately detects binding sites for both ornithine and G418 (geneticin), an aminoglycoside antibiotic. In contrast to chloroquine and ivermectin, both compounds bind preferably to the hydrophilic sites on the surface of ODC (Figure 10b–d). If applied to treat COVID-19, the drug eflornithine should bind to similar sites on the surface of COVID-19 proteins (hydrophobic and kinetically active, i.e., stable).

With the expected number of targets between 10% and 15% of the total number of residues, which corresponds to the fastest normal mode, the SAGNM algorithm recognized the residues Asp-44, Ala-281, and Phe-284 of the chain A of 1njj as kinetically hot. The corresponding predictions are Thr-21, Phe-40, Phe-41, Val-42, Ala-43, Asp-44, Leu-45, Gly-46, Asp-47, Ile-48, Gly-240, Thr-241, Arg-277, Tyr-278, Tyr-279, Val-280, Ala-281, Ser-282, Ala-283, Phe-284, Thr-285, Leu-286, Ala-287, Val-288, Glu-384, Asp-385, Met-386, Gly-387, Ala-388, Tyr-407, Val-408, Val-409, and Ser-410. See Figure S8 in the Supplementary Materials for their distribution.

### 3.7. Spike Glycoproteins and Their Interactions

#### 3.7.1. ACE2 Binding Patterns to SARS and COVID-19 Spike Glycoproteins

**Figure 8:**
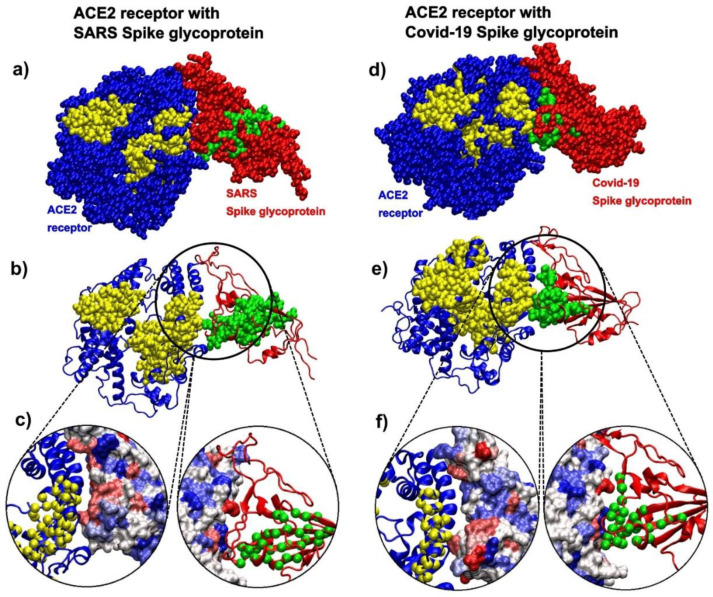
SARS spike glycoprotein chain B RBD bound to the angiotensin-converting enzyme 2 (ACE2) receptor (pdb id 6cs2) in comparison to COVID-19 spike glycoprotein chain A RBD bound to the ACE2 receptor (pdb id 6m0j). (**a**) The ACE2 receptor is represented via blue atoms and its SAGM predictions are yellow atoms. SARS spike glycoprotein is represented via red atoms, and its SAGNM predictions and green atoms. (**b**) The ACE2 receptor is represented as a blue ribbon, and its SAGM predictions are yellow atoms. SARS spike glycoprotein is the red ribbon, and its SAGNM predictions and green atoms. (**c**) Contact areas for both chains are represented as hydrophobicity surfaces. The contact chains in each case are shown as ribbons, and predictions are represented via Cα atoms only. (**d**) The ACE2 receptor is represented via blue atoms, and its SAGM predictions are yellow atoms. COVID-19 spike glycoprotein is represented via red atoms, and its SAGNM predictions and green atoms. (**e**) The ACE2 receptor is represented as a blue ribbon, and its SAGM predictions are yellow atoms. COVID-19 spike glycoprotein is the red ribbon, and its SAGNM predictions are green atoms. (**f**) Contact areas for both chains are represented as hydrophobic surfaces. The contact chains in each case are shown as ribbons, and predictions are represented via Cα atoms only.

The analysis of the contact patterns between the ACE2 receptor and the spike glycoprotein receptor binding domains (RBD) in SARS (pdb id 6cs2 [70]) and SARS-CoV-2 (pdb id 6m0j [7]) reveals a difference in the distribution of kinetically active residues important for binding between RBD and ACE2 (Figure 8). The conformationally stable SARS-RBD has a smaller number of kinetically active and adjoining residues (SAGNM predictions) in direct contact with ACE2 (Figure 8a–c), while SAGNM predictions in COVID-19 RBD are directly oriented and are in contact with the active residues in ACE2 (Figure 8d–f). In SARS, active residues are mostly perpendicular to the interfacial plane (compare the distributions of Cα atoms in Figure 8a,d). This should make the binding affinity between the COVID-19-RBD and the ACE2 receptor stronger than between the SARS-RBD and the ACE2 receptor. In both cases, the predicted residues are recognized via the fastest vibrational mode [43]. For 6cs2, the expected number of targets was between 22% and 25%, and this corresponds to the first, fastest mode for SARS spike glycoprotein (chain B), and the fastest six modes for ACE2 (chain D). For 6m0j, the expected number of targets was between 20% and 22%, and this also corresponds to the first, fastest mode for the COVID-19 spike glycoprotein receptor-binding domain (chain E), and the fastest six modes for ACE2 (chain A). For the full list of hot residues and predictions for both cases, see Figure S9 in the Supplementary Materials and the list below it.

#### 3.7.2. SARS-CoV Spike Glycoprotein and Glycans

**Figure 9:**
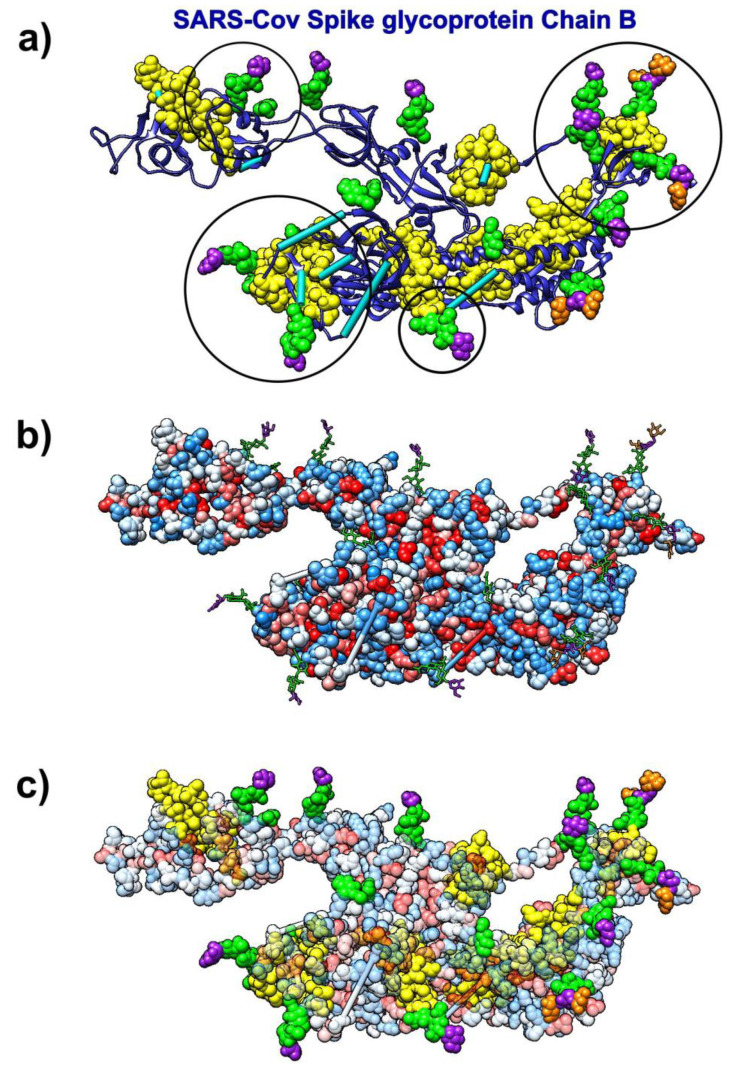
SARS-CoV spike glycoprotein (Chain B, pdb id 6nb6) with glycans (NAG, BMA, MAN) bound to it. (**a**) Ribbon-like representation of SARS spike glycoprotein. The SAGNM predictions are yellow atoms. BMA molecules are represented via purple atoms. MAN molecules are represented via orange atoms. NAG molecules are represented via green atoms. Cyan bars represent missing glycoprotein segments. Circles represent areas where the SAGNM predictions recognize real binding spots. (**b**) SARS spike glycoprotein is depicted via hydrophobicity colored atoms. Glycans (NAG, BMA, MAN) are represented via colored bonds (same colors as above). (**c**) SARS spike glycoprotein is depicted via transparent hydrophobicity colored atoms. Glycans (NAG, BMA, MAN) are represented via colored bonds (same colors as above). The SAGNM predictions are yellow atoms. Glycans (NAG, BMA, MAN) are represented via colored atoms.

The analysis of kinetically active and adjoining residues in the SARS-CoV spike glycoprotein monomer (pdb id 6nb6) reveals that they are attractive binding spots for glycans (Figure 9). Glycans form the glycan shield, which was already suggested to assist in immune evasion similarly to the HIV-1 envelope trimer [72]. The kinetically active residues recognized by the SAGNM algorithm [43] can be used as target areas for drugs aimed at removing/disrupting the viral glycan shield. Those residues are not particularly hydrophobic and should be targeted by drugs that bind to hydrophilic patches, and have complementary charges.

#### 3.7.3. SARS Spike Glycoprotein RBD and Human Antibody Fragment

**Figure 10:**
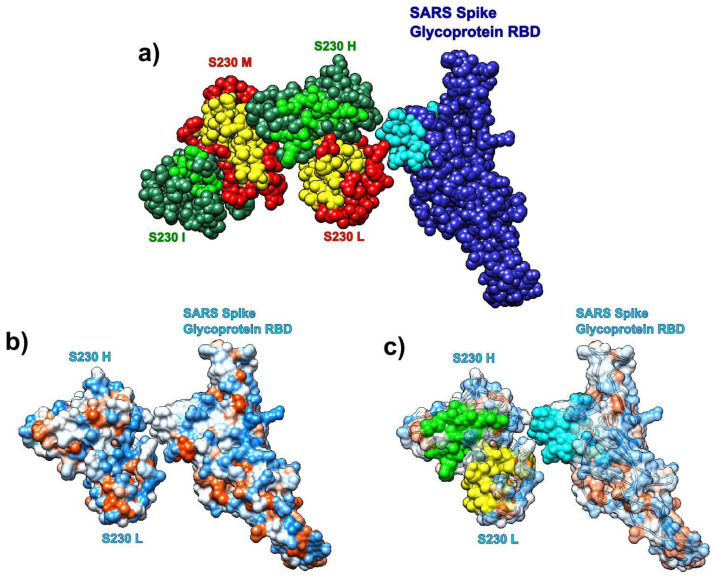
Receptor binding domain (RBD) of SARS-CoV spike glycoprotein (chain A, pdb id 6nb6) with the human neutralizing S230 antibody FAB fragment. (**a**) SARS-CoV RBD (blue, chain A) with heavy (green, H, and I) and light (red, L, and M) chains. Predictions are cyan (SARS), yellow (S230 light), and light green (S230 heavy). (**b**) Hydrophobic surface of SARS RBD bound to S230 (chains H and L). (**c**) Transparent hydrophobic surface of SARS RBD and S230 (chains H and L) with predictions.

We also analyzed the distribution of SAGNM recognized residues in the structure formed by the SARS spike glycoprotein RBD and the human neutralizing S230 antigen-binding fragment (FAB) (pdb id 6nb6). The analysis reveals that the S230 antibody binds to kinetically active residues in SARS RBD, while heavy and light chains in S230 communicate via kinetically active residues (see Figure 10). The binding residues are mostly neutral to hydrophilic; thus, any potential drug should be able to bind to similar surfaces (neutral/hydrophilic and stable). For the list of hot residues and predictions, see Figure S10 in the Supplementary Materials and the list below it.

## 4. Discussion and Conclusions

COVID-19 is the first modern, severe global pandemic caused by a coronavirus, and there are no guarantees that it will be the last. Our society needs not only to develop an effective and efficient treatment for the current disease but also has to have a set of protocols and standards to promptly address all future, similar pandemics. In this manuscript, we presented our strategy to recognize potential drug-binding residues in human and viral proteins. We analyzed six currently approved drugs (chloroquine, ivermectin, remdesivir, sofosbuvir, boceprevir, and eflornithine). Our results indicate that small, drug-like compounds preferentially bind to kinetically active and adjoining residues, and thus seek stable residues characterized by fast normal modes with small amplitude fluctuations [43]. Some of the drugs we analyzed preferentially seek active patches that are hydrophobic (chloroquine, ivermectin), while others prefer hydrophilic surfaces (remdesivir, sofosbuvir, eflornithine). We can postulate that in a water environment, drugs that bind to hydrophilic patches will be more stable, as their removal will lead to the reduction in structural entropy, but a full account of this proposition will require calculations of binding free energy differences using, for instance, still numerically expensive molecular dynamics simulations [73,74,75,76]. We can also propose that the drugs/small molecules that bind to deep pockets will be more stable, and thus more effective. Our algorithm accurately recognizes such pockets as binding spots for drugs (Figure 1a, Figure 3, and Figure 10), and small peptides (see, in particular, Figure 6a in [43]).

Multidrug cocktails are frequently used to treat viral diseases [77]. Our analysis shows that in designing antiviral drug cocktails, the binding affinity between drugs and kinetically active (stable) sites should be combined with the information on their hydrophobic and hydrophilic properties in an attempt to avoid binding competition, increase drug cocktail efficiency, and reduce toxicity and other unwanted side effects.

Our results are concordant with full atom docking and simulations studies [13,14,15,16,17,18,19] that emphasized sofosbuvir, remdesivir, hydroxychloroquine, and ivermectin, compounds that we also analyzed. This indicates that protein–ligand docking is a multistep process, guided both by coarse-grained properties of a bigger binding partner, and detailed, atomic-scale properties of the binding pocket and a small ligand.

In our analysis, we used both viral–parasitic, as well as human proteins. The analysis shows that kinetically active residues exist in both human and non-human proteins/enzymes and that drugs bind indiscriminately to them regardless of their origin. The compounds that bind to human proteins potentially offer longer-lasting treatments as host cells and tissues have less chance of developing drug resistance through single point mutations.

The procedure we described here is fast and effective and can analyze a protein structure much faster than computationally more demanding docking or molecular dynamics simulations, with complex multistep pipelines [78,79,80,81]. It is based on the assumption that proteins do not experience significant conformational changes upon ligand binding, which is often the case when binding spots are hydrophobic [61]. Its advantage is not in its efficiency, but also in its ability to suggest general binding patterns between proteins and drugs or small peptides. It can be used to filter binding areas on protein surfaces and thus facilitate preclinical stages in drug design. Binding spots in various proteins can be very effectively predicted with our SAGNM approach and accessed with other bioinformatics tools for charge and shape complementarity, exposed surface area, binding affinity, atomic mass, and other properties as well. However, the SAGNM algorithm has its limitations. It predicts binding areas in relatively broad strokes. Additional tools able to filter out residues with relatively small surface accessible areas, and/or with incompatible charge and hydrophobic properties to the ligands of interest could improve the prediction. Additionally, the SAGNM algorithm cannot determine binding free energies or binding orientations of small molecules. For that aim, other docking tools or molecular dynamics studies should be applied, as explained above.

The SAGNM procedure is often not effective with homodimers or with protein complexes formed of similarly sized protein chains [43]. When two molecules of different sizes form a complex, the larger partner, i.e., the protein, does not have to significantly change its conformation during binding to accommodate smaller ligand [61]. This preserves its contact map (matrix **Γ**) and the distribution of fast modes and hot residues. However, when two proteins of similar sizes interact, they may rearrange their conformations simply through their sheer size, and thus interrupt their contact maps (**Γ** matrices). See Figure 1, Figure 4, Figure 5, and Figure 6c in [43] for the analysis and statistics of cases where the SAGNM fails. Therefore, the SAGNM approach is primarily aimed at binding residues recognition in cases where the binding partner is a small compound or small peptide. Its effectiveness can be improved by combining its output with other tools. Therefore, it can be used as a step in complex docking and simulation pipelines.

We envision the SAGNM procedure as the first step in a ligand docking and free energy simulation pipeline. It can suggest an area on the surface of the target protein where potential drugs should bind. The next steps will limit their calculation to that area only. If the area suggested by the SAGNM is a tenth of the protein’s surface area, this means that all subsequent computational costs are reduced accordingly. The cost of SAGNM and surface area calculations is negligible in comparison. Therefore, the approach based on the SAGNM algorithm offers an effective and efficient method to speed up preclinical in silico stages in structural drug design.

Recent advances in machine learning helped advance our ability to predict and design protein structures [82], but the full theoretical foundations for protein folding and binding is still lacking. The quality of the machine learning protocol directly depends on the quality and size of training datasets and, thus, in many ways follows classical methods based on statistical potentials and homology modeling [83,84]. Our results can also help in that respect as they offer interpretation on how residue packing inside protein segments guides their assemblage.

The results depicted here show that in proteins that interact with small, drug-like molecules contacting scaffolds are defined by kinetically hot and residues surrounding them, regardless of the nature of the small ligand, assuming that the protein structure does not change significantly after binding. A similar conclusion related to protein–protein interactions was given in [43]. As we showed above, the full binding behavior cannot be accessed through the analysis of kinetically active residues and their neighbors only. The full atom analysis is still required for the detailed assessment of protein–drug binding. The coarse-grained analysis (SAGNM algorithm) thus perceives only the outline of the binding funnel, while a full atom analysis (docking and binding free energy studies) grasps finer patterns inside that outline. This approach should in principle be similar to the current improvements in deep neural network (DNN) architectures aimed at image recognition and classification (Brendel and Bethge [85]). The improvement is based on splitting images into small local image features (e.g., outlines) without taking into account their spatial ordering, a strategy closely related to the pre deep-learning bag-of-features (BoF) models [86]. The image classification improvement stems from the observation that standard DNN architectures perceive images primarily through textures, as opposed to human perception, which is primarily based on the outlines and shapes of objects [87]. If we translate this to the problem of protein–ligand binding, we can say that the outline is determined primarily by the protein and the packing of its residues, and fine binding features (“binding textures”) stem from the joint properties of the smaller binding partner and the binding pocket of the protein. In this sense, the SAGNM approach is similar to human vision, and molecular docking and dynamics studies to the machine, DNN-based vision. This observation opens a space for further work, where the molecular binding will be treated as a two-step process where the coarse-grained shape of a binding funnel will be determined by the larger partner in the first step, and the final binding position and orientation by the multiple and detailed features of the binding funnel and a smaller partner inside that funnel.

## Figures and Tables

**Table 1:** Comparison of existing drugs currently being tested for the antiviral treatment and prevention of COVID-19 through drug repurposing.

Drug	Indication	Dosage in Individuals Aged ≥ 12 Years	Effectiveness	Side Effects	Precautions in Patients with Complications			
					Cardio-Pulmonary	Renal	Hepatic	Retinal[M1]
Chloroquine	Treatment Prevention	500–600 mg weekly	Malaria, Amebiasis, Porphyria Cutanea Tarda	Serious	Yes	Yes	Yes	Yes
Ivermectin	Treatment Prevention	3–15 mg once	Parasitic infestations	Mild/Serious	No	Yes	Yes	No
Remdesivir	Treatment	100–200 mg daily	Ebola, Marburg virus diseases	Mild	No	Yes	No	No
Sofosbuvir	Treatment	400 mg daily	Hepatitis-C, HIV	Mild/Moderate	Yes	Yes	Yes	Yes
Boceprevir	Treatment	200 mg daily	Hepatitis-C	Mild/Serious	Yes	No	Yes	Yes
α-Difluoromethylornithine	Treatment	300–400 mg/kg/day, cream	Trypanosomiasis, reduction of facial hair in women	Mild/Serious	No	No	Yes	No

## Supplementary Materials

The following are available online at https://www.mdpi.com/2218-273X/10/9/1346/s1, Figure S1: Hot residues and contact predictions for Lactate Dehydrogenase and Saposin B, determined by SAGNM, Figure S2: Ivermectin and its target protein, human glycine receptor Alpha-3 (pdb id 5vdh) with hot 24 residues and contact predictions determined by SAGNM, Figure S3: Ivermectin and its target protein, C. elegans glycine receptor Alpha-3 (pdb id 3rif), Figure S4: Remdesivir bound to the primer RNA inside the central channel of SARS-COV-2 RNA 83 dependent RNA polymerase (RdRp), NSP12) (pdb id 7bv2), Figure S5: Sofosbuvir and its target protein Hepatitis C virus (HCV) RdRp (chain A in pdb id 4wtg) 117 with hot residues and contact predictions determined by SAGNM, Figure S6: Covid-19 RNA directed RNA polymerase with cofactors NSP7 and NSP8 (pdb id 6m71), Figure S7: Hot residues and predictions for the SARS-Cov-2 main protease bound to boceprevir (pdb id 6wnp), Figure S8: Hot residues and predictions for ornithine decarboxylase (pdb id 1njj) chain A, Figure S9: Hot residues and predictions for the ACE2 receptor with SARS-COV Spike glycoprotein (pdb id 6cs2, left) and the ACE2 receptor with Covid-19 Spike glycoprotein (pdb id 6m0j, right), Figure S10: Hot residues and predictions for the receptor binding domain (RBD) of SARS-COV spike glycoprotein (Chain A, pdb id 6nb6) with human neutralizing S230 antibody FAB fragment.
