# Supplementary material for: Recognition of Potential COVID-19 Drug Treatments through the Study of Existing Protein–Drug and Protein–Protein Structures: An Analysis of Kinetically Active Residues
Source: Biomolecules. 2020 Sep 21;10(9):1346. doi: 10.3390/biom10091346 (PMC7565175; doi:10.3390/biom10091346)
Supplement: Supplementary file 1 [file biomolecules-10-01346-s001.pdf]

## Supplementary Material Figures

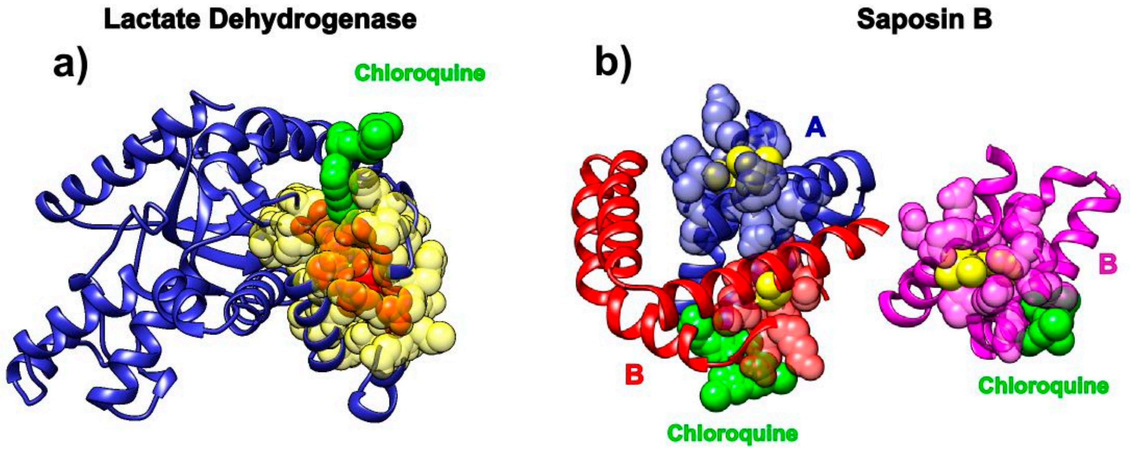


**Figure S1.** Hot residues and contact predictions for Lactate Dehydrogenase and Saposin B, determined by SAGNM. Images on the left depict chloroquine bound to the cofactor binding site of *Plasmodium Falciparum* Lactate Dehydrogenase (pdb id 1cet). Images on the right depict chloroquine bound to Saposin B (pdb id 4v2o). (**a**) Lactate Dehydrogenase is depicted as a blue ribbon, SAGNM predictions are transparent yellow atoms and hot residues are red atoms. Chloroquine is depicted as green atoms. (**b**) Saposin B chains are depicted as blue (chain A), pink (chain B), and red (chain C) ribbons. SAGNM predictions are depicted as transparent blue, pink, and red atoms, and hot atoms are depicted as yellow atoms. Chloroquine molecules are depicted via green atoms.

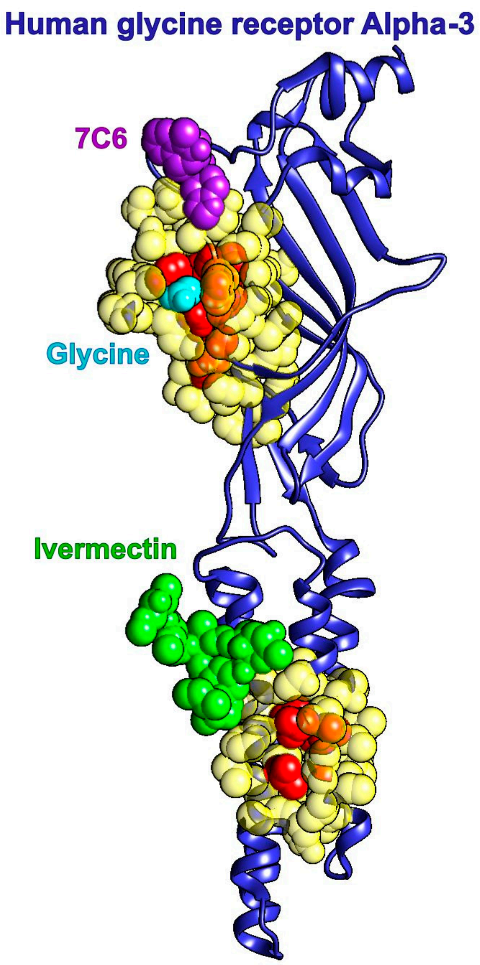


**Figure S2.** Ivermectin and its target protein, human glycine receptor Alpha-3 (pdb id 5vdh) with hot residues and contact predictions determined by SAGNM. Chain A from human glycine receptor Alpha-3 is represented as a blue ribbon. SAGNM predictions are depicted as transparent yellow atoms and hot residues are red atoms. Ivermectin is represented via green atoms. The glycine molecule is represented as cyan atoms. 7C6 molecule is represented as purple atoms.

The SAGNM algorithm recognized the residues Glu-157, Ser-158, Phe-168, Phe-207, Thr-208, Cys-209, Ile-210, Glu-211, Ser-238, Gly-256, Thr-259, Val-260, Val-294, and Leu-298 as from the chain A from 5vdh.pdb as kinetically hot. Their influence is spread to the residues Asn-38, Val-39, Thr-40, Cys-41, Pro-96, Asp-97, Leu-98, Phe-99, Phe-100, Ala-101, Ile-153, Met-154, Gln-155, Leu-156, Glu-157, Ser-158, Phe-159, Gly-160, Tyr-161, Thr-162, Met-163, Asn-164, Asp-165, Leu-166, Ile-167, Phe-168, Glu-169, Trp-170, Gln-171, Asp-172, Leu-195, Arg-196, Tyr-197, Cys-198, Thr-199, Lys-200, His-201, Asn-203, Thr-204, Gly-205, Lys-206, Phe-207, Thr-208, Cys-209, Ile-210, Glu-211, Val-212, Arg-213, Phe-214, His-215, Ile-234, Val-235, Ile-236, Leu-237, Ser-238, Trp-239, Val-240, Ser-241, Phe-242, Arg-252, Val-253, Ala-254, Leu-255, Gly-256, Ile-257, Thr-258, Thr-259, Val-260, Leu-261, Thr-262, Met-263, Thr-264, Cys-290, Leu-291, Leu-292, Phe-293, Val-294, Phe-295, Ser-296, Ala-297, Leu-298, Leu-299, Glu-300, Tyr-301, and Ala-302.

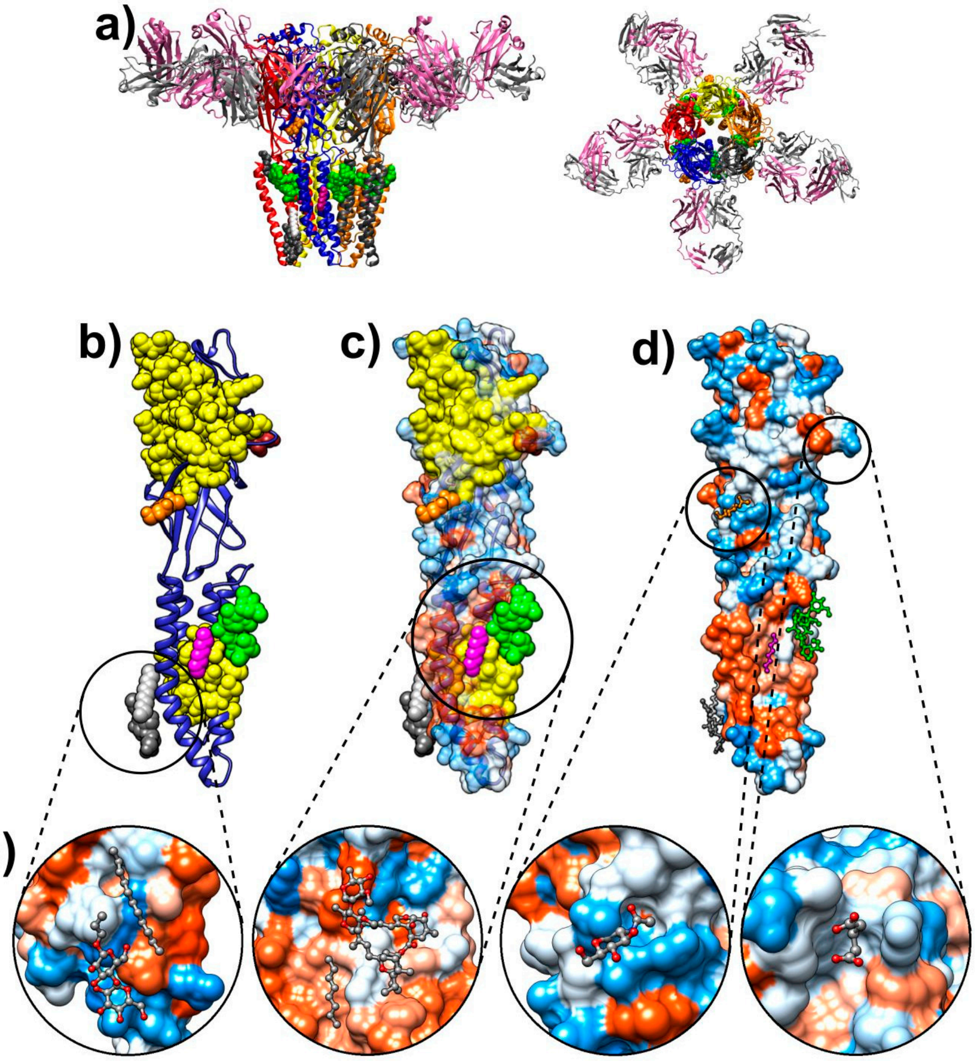


**Figure S3.** Ivermectin and its target protein, *C. elegans* glycine receptor Alpha-3 (pdb id 3rif). (**a**) Five pentamer chains (A to E) are represented as ribbons. Light chains from mouse monoclonal FAB fragment, represented as pink ribbons (chains K to O), and heavy chains (chains F to J) represented as grey ribbons. Ivermectin is represented via green spherical atoms. Glycine molecules are brown and represented via spherical atoms. OCT (N-octane) is represented as a pink molecule, and NAG (2-acetamido-2-deoxy-beta-d-glucopyranose) as an orange molecule. UND (undecane) and LMT (dodecyl-beta-d-maltoside) as light and dark grey molecules respectively. Left - side view, right – top view. (**b**) Chain B from *C. elegans* glycine receptor Alpha-3 is represented as a blue ribbon. SAGNM predictions are depicted as yellow atoms. Ivermectin is represented via green spherical atoms. Glycine molecules are brown and represented via spherical atoms. OCT (N-octane) is represented as a pink molecule, and NAG (2-acetamido-2-deoxy-beta-d-glucopyranose) as an orange molecule. UND (undecane) and LMT (dodecyl-beta-d-maltoside) as light and dark grey molecules respectively. (**c**) Chain B from *C. elegans* glycine receptor Alpha-3 is depicted as a transparent hydrophobic surface. SAGNM predictions are yellow. (**d**) Chain A from *C. elegans* glycine receptor Alpha-3 is depicted as an opaque hydrophobic surface. The ligands are represented as ball-and-sticks. (**e**) The four insets show Ivermectin, Glycine, OCT, NAG, UND, and LMT ligands inside the pockets on the surface of the chain B of *C. elegans* glycine receptor Alpha-3. The figure is produced with the VMD and UCSF Chimera programs.

The expected number of predictions for the SAGNM algorithm was set to be between 33% and 34%, and that corresponds to 3 fastest modes, as with the human glycine receptor alpha-3 (pdb id 5vdh analyzed in the main text). The SAGNM algorithm recognized the residues Val-32, Met-34, Glu-57, Ile-112, Asn-114, Tyr-160, Cys-202, Ser-231, Gly-249, Leu-253, Ile-287, and Leu-291 from chain A

from 3rif structure as hot. Their influence is spread to the residues Val-28, Val-29, Val-30, Ser-31, Val-32, Asn-33, Met-34, Leu-35, Leu-36, Arg-37, Thr-38, Leu-53, Thr-54, Leu-55, Arg-56, Glu-57, Ser-58, Trp-59, Ile-60, Asp-61, Leu-64, Ser-65, Tyr-66, Gly-67, Val-68, Lys-69, Gly-70, Gln-73, Pro-74, Asp-75, Phe-76, Val-77, Ile-78, Leu-79, Val-108, Leu-109, Ile-110, Arg-111, Ile-112, His-113, Asn-114, Asp-115, Gly-116, Thr-117, Val-118, Leu-119, Tyr-120, Ser-121, Asp-147, Leu-148, Ala-149, Ser-150, Lys-156, Asp-157, Ile-158, Glu-159, Tyr-160, Leu-161, Trp-162, Lys-163, Glu-164, Pro-167, Leu-168, Gln-169, Tyr-190, Cys-191, Thr-192, Ser-193, Gly-198, Ile-199, Tyr-200, Ser-201, Cys-202, Leu-203, Arg-204, Thr-205, Thr-206, Leu-227, Val-228, Ile-229, Val-230, Ser-231, Trp-232, Val-233, Ser-234, Phe-235, Arg-245, Val-246, Thr-247, Leu-248, Gly-249, Val-250, Thr-251, Thr-252, Leu-253, Leu-254, Thr-255, Met-256, Thr-257, Cys-283, Met-284, Thr-285, Phe-286, Ile-287, Phe-288, Cys-289, Ala-290, Leu-291, Leu-292, Glu-293, Phe-294, and Ala-295.

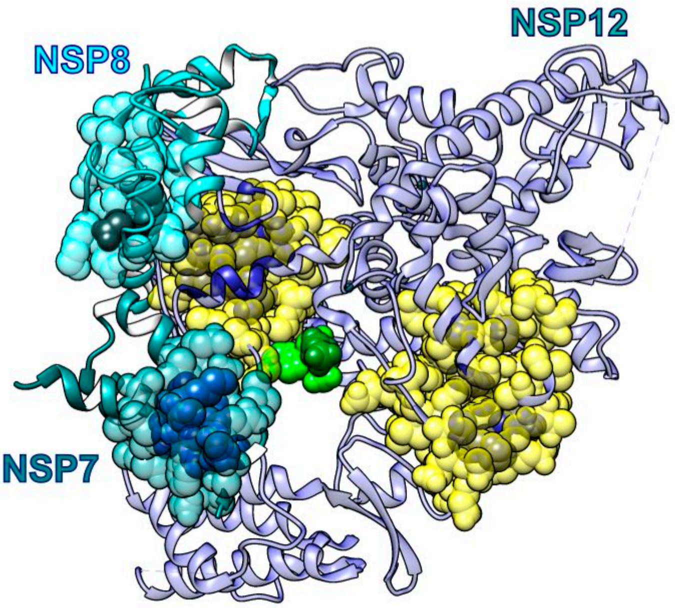


**Figure S4.** Remdesivir bound to the primer RNA inside the central channel of SARS-COV-2 RNA dependent RNA polymerase (RdRp), NSP12) (pdb id 7bv2). a) Three RNA polymerase chains, NSP 12, NSP7, and NSP8, are represented as blue, cyan, and dark cyan ribbons. Remdesivir is represented via green atoms and pyrophosphate as dark green atoms. SAGNM predictions for NSP12 are depicted as transparent yellow atoms and hot residues are blue atoms. SAGNM predictions for NSP7 are transparent green atoms and hot residues are blue atoms. SAGNM predictions for NSP9 are transparent cyan atoms and hot residues are gray atoms. Remdesivir is represented via green atoms and pyrophosphate as dark green atoms.

The SAGNM algorithm recognized the residues Gly-503, Thr-538, Ile-539, Thr-540, Gln-541, Ala-558, Val-560, Ser-561, Val-609, His-613, Glu-665, Met-666, Val-667, Met-668, Ala-702, Ala-706, Phe-753, Cys-765, and Asn-767 in chain A as hot, the residues Asp-161 and Ile-185 in chain B as hot, and the residues Lys-7, Ser-10, His-36, Ile-39, Ala-48, and Lys-51 in chain C as hot. The influence of the hot residues in chain A is spread to the residues Ala-376, Asp-377, Pro-378, Asp-499, Lys-500, Ser-501, Ala-502, Gly-503, Phe-504, Pro-505, Phe-506, Asn-507, Asn-534, Val-535, Ile-536, Pro-537, Thr-538, Ile-539, Thr-540, Gln-541, Met-542, Asn-543, Leu-544, Lys-545, Ala-554, Arg-555, Thr-556, Val-557, Ala-558, Gly-559, Val-560, Ser-561, Ile-562, Cys-563, Ser-564, Thr-565, Val-605, Tyr-606, Ser-607, Asp-608, Val-609, Glu-610, Asn-611, Pro-612, His-613, Leu-614, Met-615, Gly-616, Trp-617, Cys-659, Ala-660, Gln-661, Val-662, Leu-663, Ser-664, Glu-665, Met-666, Val-667, Met-668, Cys-669, Gly-670, Gly-671, Ser-672, Leu-673, Tyr-674, Val-675, Lys-676, Ser-681, Ser-682, Gly-683, Asp-684, Gln-698, Ala-699, Val-700, Thr-701, Ala-702, Asn-703, Val-704, Asn-705, Ala-706, Leu-707, Leu-708, Ser-709, Thr-710, Tyr-748, Leu-749, Arg-750, Lys-751, His-752, Phe-753, Ser-754, Met-755, Met-756, Ile-757, Asp-761, Ala-762, Val-763, Val-764, Cys-765, Phe-766, Asn-767, Ser-768, Thr-769, Tyr-770, Ala-771, Gly-774,

Leu-775, Val-776, Ala-777, Ser-778, Asn-781, Phe-782, Val-785, Thr-801, Glu-802, Thr-803, Asp-804 and Leu-805. The influence of the hot residues in chain B is spread to the residues Val-130, Val-131, Gln-157, Gln-158, Val-159, Val-160, Asp-161, Ala-162, Asp-163, Ser-164, Lys-165, Ala-181, Trp-182, Pro-183, Leu-184, Ile-185, Val-186, Thr-187, Ala-188 and Leu-189. And the influence of the hot residues in chain C is spread to the residues Ser-4, Asp-5, Val-6, Lys-7, Cys-8, Thr-9, Ser-10, Val-11, Val-12, Leu-13, Val-33, Gln-34, Leu-35, His-36, Asn-37, Asp-38, Ile-39, Leu-40, Leu-41, Ala-42, Thr-45, Thr-46, Glu-47, Ala-48, Phe-49, Glu-50, Lys-51, Met-52, Val-53, and Ser-54.

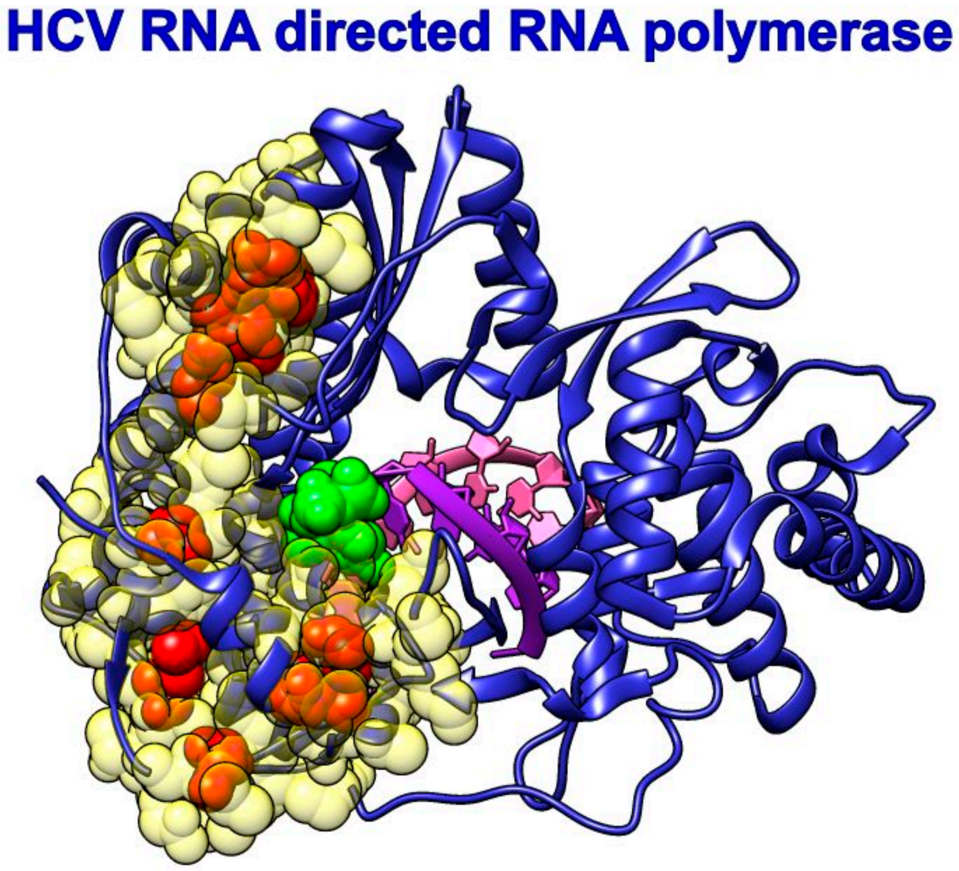


**Figure S5.** Sofosbuvir and its target protein Hepatitis C virus (HCV) RdRp (chain A in pdb id 4wtg) with hot residues and contact predictions determined by SAGNM. Chain A from HCV RdRp is represented as a blue ribbon. SAGNM predictions are depicted as transparent yellow atoms and kinetically hot residues are red atoms. Sofosbuvir is represented via green atoms. RNAs are pink and purple ribbons.

The SAGNM algorithm recognized the residues Ser-3, Tyr-4, Ala-9, Leu-10, Ile-11, Thr-12, Pro-13, Cys-14, Thr-40, Thr-41, Ser-42, Ser-44, Ala-45, Arg-48, Tyr-64, Pro-135, Thr-136, Thr-137, Ile-138, Met-139, Ala-140, Lys-141, Asn-142, Glu-143, Gly-153, Lys-154, Lys-155, Pro-156, Ala-157, Arg-158, Leu-159, Ile-160, Val-161, Asp-225, Ser-226, Thr-227, Val-228, Thr-229, Glu-230, Tyr-261, Val-262, Gly-263, Gly-264, Pro-265, Met-266, Phe-267, Asn-268, Ser-269, Lys-270, Gly-271, Gln-272, Thr-273, Cys-274, Gly-275, Tyr-276, Arg-277, Arg-278, Cys-279, Arg-280, Ala-281, Ser-282, Gly-283, Thr-294, Cys-295, Tyr-296, Val-297, Lys-298, Ala-299, Leu-300, Ala-301, Ala-302, Cys-303, Ala-305, Ala-306, Asn-335, Leu-336, Arg-337, Ala-338, Phe-339, Thr-340, Glu-341, Ala-342, Met-343, Thr-344, Arg-345, Tyr-346, Ser-347, Ala-348, and Pro-349 from 4wtg.pdb as predictions.

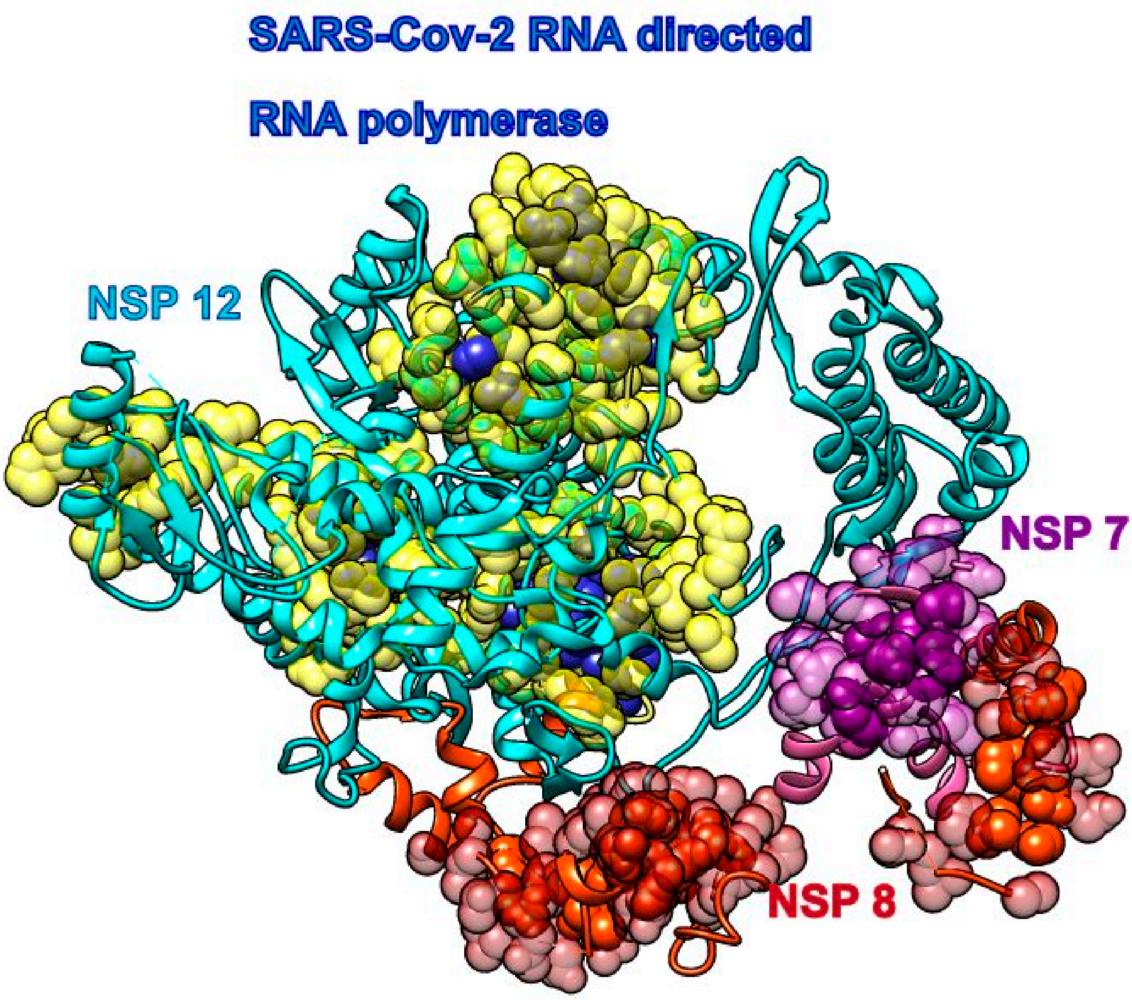


**Figure S6.** Covid-19 RNA directed RNA polymerase with cofactors NSP7 and NSP8 (pdb id 6m71). The NSP 12 chain is cyan, its SAGNM predictions are transparent yellow atoms, and hot residues are opaque blue atoms. The NSP 7 chain is pink, its SAGNM predictions are transparent purple atoms and hot residues are opaque purple atoms. The NSP 8 chain is orange, it SAGNM predictions are transparent red atoms, and its hot atoms opaque orange atoms. The dashed lines represent segments missing from the coordinates file.

The SAGNM algorithm recognized the residues Gly-230, Cys-298, Cys-310, Asn-314, Gly-352, Ala-502, Gly-503, Thr-538, Ile-539, Thr-540, Gln-541, Gly-559, Val-560, Ser-561, Val-609, Glu-665, Val-667, Met-668, Ala-702, Ala-706, Val-763, Cys-765, and Asn-767 from the chain A from 6m71 as hot.

It also recognized the residues Met-196, Arg-197, Asn-198, Ala-199, Gly-200, Ile-201, Val-202, Thr-225, Thr-226, Pro-227, Gly-228, Ser-229, Gly-230, Val-231, Pro-232, Val-233, Val-234, Asp-274, Thr-276, Arg-279, Tyr-294, His-295, Pro-296, Asn-297, Cys-298, Val-299, Asn-300, Cys-301, Leu-302, Cys-306, Ile-307, Leu-308, His-309, Cys-310, Ala-311, Asn-312, Phe-313, Asn-314, Val-315, Leu-316, Phe-317, Ser-318, Gly-327, His-347, Phe-348, Arg-349, Glu-350, Leu-351, Gly-352, Val-353, Val-354, His-355, Asn-356, Ala-376, Asp-377, Pro-378, Leu-498, Asp-499, Lys-500, Ser-501, Ala-502, Gly-503, Phe-504, Pro-505, Phe-506, Asn-507, Gly-510, Lys-511, Ala-512, Asn-534, Val-535, Ile-536, Pro-537, Thr-538, Ile-539, Thr-540, Gln-541, Met-542, Asn-543, Leu-544, Lys-545, Arg-555, Thr-556, Val-557, Ala-558, Gly-559, Val-560, Ser-561, Ile-562, Cys-563, Ser-564, Thr-565, Val-605, Tyr-606, Ser-607, Asp-608, Val-609, Glu-610, Asn-611, Pro-612, His-613, Leu-614, Met-615, Gly-616, Trp-617, Cys-659, Ala-660, Gln-661, Val-662, Leu-663, Ser-664, Glu-665, Met-666, Val-667, Met-668, Cys-669, Gly-670, Gly-671, Ser-672, Leu-673, Tyr-674, Val-675, Lys-676, Ser-681, Gly-683, Asp-684, Gln-698, Ala-699, Val-700, Thr-701, Ala-702, Asn-703, Val-704, Asn-705, Ala-706, Leu-707, Leu-708, Ser-709, Thr-710, Lys-751, His-752, Phe-753, Ser-754, Met-755, Met-756, Ile-757, Ser-759, Asp-760, Asp-761, Ala-762, Val-763, Val-764,

Cys-765, Phe-766, Asn-767, Ser-768, Thr-769, Tyr-770, Ala-771, Gly-774, Leu-775, Val-776, Ala-777, Ser-778, Asn-781, Phe-782, Val-785, Pro-809, and Phe-812 as predictions.

The SAGNM algorithm recognized the residues Ile-132, Trp-154, Ile-156, Val-159, Asp-161, Leu-184, Ile-185, Val-186, Thr-187, and Ala-188 from chain B from 6m71 as hot.

It also recognized the residues Lys-127, Leu-128, Met-129, Val-130, Val-131, Ile-132, Pro-133, Asp-134, Tyr-135, Asn-136, Thr-137, Tyr-138, Thr-146, Phe-147, Thr-148, Ala-150, Ser-151, Ala-152, Leu-153, Trp-154, Glu-155, Ile-156, Gln-157, Gln-158, Val-159, Val-160, Asp-161, Ala-162, Asp-163, Ser-164, Lys-165, Val-167, Leu-180, Ala-181, Trp-182, Pro-183, Leu-184, Ile-185, Val-186, Thr-187, Ala-188, Leu-189, Arg-190, and Ala-191 as predictions.

The SAGNM algorithm recognized the residues Val-6, Ser-10, Leu-35, Ile-39, Ala-48, and Met-52 from chain C from 6m71 as hot.

It also recognized the residues Met-3, Ser-4, Asp-5, Val-6, Lys-7, Cys-8, Thr-9, Ser-10, Val-11, Val-12, Leu-13, Cys-32, Val-33, Gln-34, Leu-35, His-36, Asn-37, Asp-38, Ile-39, Leu-40, Leu-41, Ala-42, Thr-45, Thr-46, Glu-47, Ala-48, Phe-49, Glu-50, Lys-51, Met-52, Val-53, Ser-54, and Leu-55 as predictions.

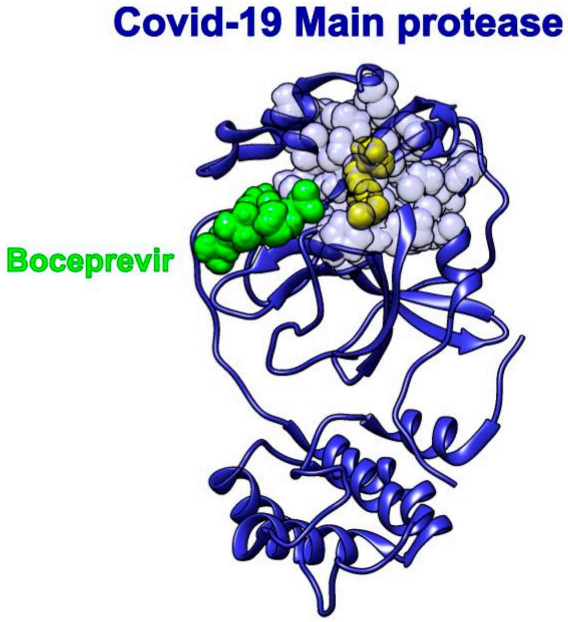


**Figure S7.** Hot residues and predictions for the SARS-Cov-2 main protease bound to boceprevir (pdb id 6wnp). The Covid-19 main protease is depicted as a blue ribbon, with SAGNM predictions as transparent blue atoms, and hot residues as opaque yellow atoms. Boceprevir is depicted as a green molecule.

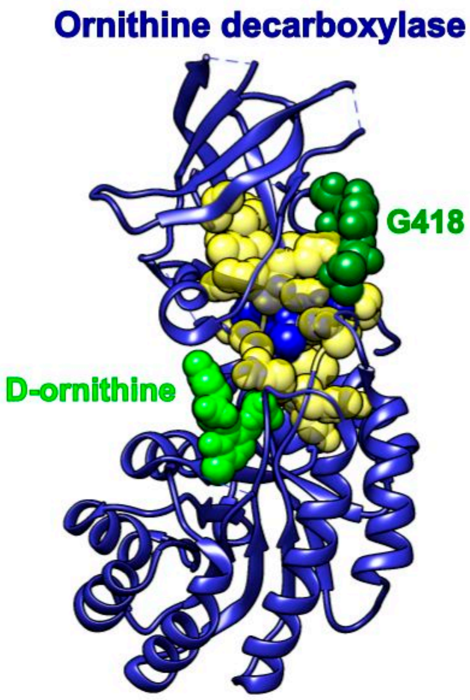


**Figure S8.** Hot residues and predictions for ornithine decarboxylase (pdb id 1njj) chain A. The chain A is depicted as a blue ribbon, with SAGNM predictions as transparent yellow atoms, and hot residues as opaque blue atoms. D-ornithine and G-418 are depicted as green and dark green molecules, respectively.

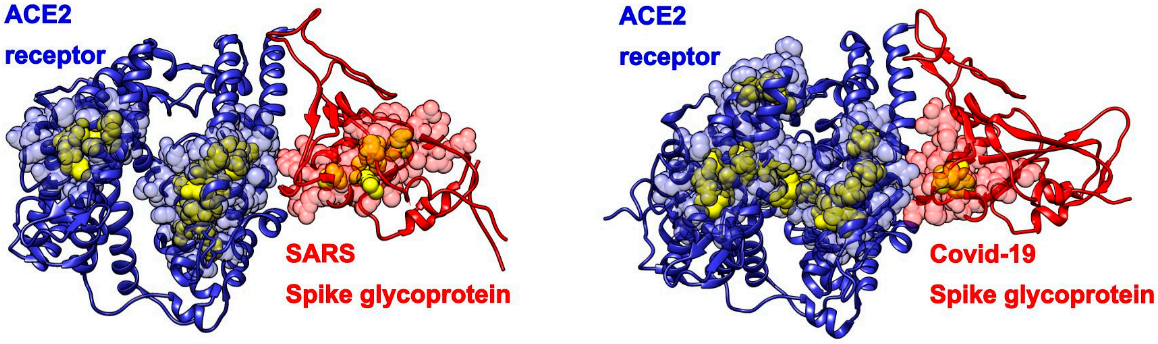


**Figure S9.** Hot residues and predictions for the ACE2 receptor with SARS-COV Spike glycoprotein (pdb id 6cs2, **left**) and the ACE2 receptor with Covid-19 Spike glycoprotein (pdb id 6m0j, **right**). Predictions are blue transparent atoms (ACE2 receptor) and red transparent atoms (SARS and Covoid-19 Spike glycoprotein). Hot residues are yellow.

The SAGNM algorithm recognized the residues Asp-385, Phe-387, Pro-493, Arg-495, and Val-496 in the chain B of 6cs2, and predictions are residues Phe-334, Pro-335, Ser-336, Val-337, Trp-340, Glu-341, Arg-342, Asn-381, Val-382, Tyr-383, Ala-384, Asp-385, Ser-386, Phe-387, Val-388, Val-389, Lys-390, Gly-391, Leu-421, Ala-422, Trp-423, Thr-425, Arg-426, Asp-429, Ile-489, Gly-490, Tyr-491, Gln-492, Pro-493, Tyr-494, Arg-495, Val-496, Val-497, Val-498, Leu-499, and Ser-500.

The SAGNM algorithm recognized the residues Ser-43, Ala-46, Tyr-50, Met-62, Met-123, Val-172, Gly-173, Leu-176, Ala-348, His-373, His-374, Glu-375, His-378, Tyr-385, Ala-403, Gly-405, Ser-409, and Cys-498 as hot in the chain D of 6cs2.

The predictions are residues Leu-39, Phe-40, Tyr-41, Gln-42, Ser-43, Ser-44, Leu-45, Ala-46, Ser-47, Trp-48, Asn-49, Tyr-50, Asn-51, Thr-52, Asn-53, Ile-54, Thr-55, Asn-58, Val-59, Gln-60, Asn-61, Met-62, Asn-63, Asn-64, Ala-65, Gly-66, Trp-69, Ile-119, Leu-120, Asn-121, Thr-122, Met-123, Ser-124, Thr-125, Ile-126, Tyr-127, Gly-130, Lys-131, Trp-168, Arg-169, Ser-170, Glu-171, Val-172, Gly-173, Lys-

174, Gln-175, Leu-176, Arg-177, Pro-178, Leu-179, Tyr-180, Phe-308, Ala-311, Ala-342, Cys-344, His-345, Pro-346, Thr-347, Ala-348, Trp-349, Asp-350, Leu-351, Gly-352, Phe-356, Arg-357, Ile-358, Leu-359, Phe-369, Leu-370, Thr-371, Ala-372, His-373, His-374, Glu-375, Met-376, Gly-377, His-378, Ile-379, Gln-380, Tyr-381, Asp-382, Met-383, Ala-384, Tyr-385, Ala-386, Ala-387, Gln-388, Pro-389, Leu-392, Arg-393, Asn-394, Gly-399, Phe-400, His-401, Glu-402, Ala-403, Val-404, Gly-405, Glu-406, Ile-407, Met-408, Ser-409, Leu-410, Ser-411, Ala-412, Ala-413, Met-474, Asp-494, Glu-495, Thr-496, Tyr-497, Cys-498, Asp-499, Pro-500, Ala-501, Ser-502, Ser-507, Asn-508, Arg-518, Thr-519, Tyr-521, Gln-522, Leu-558, Arg-559, Leu-560, and Gly-561.

The SAGNM algorithm recognized the residues Ser-43, Tyr-50, Met-62, Trp-69, Met-123, Val-172, Gly-173, Leu-176, Met-190, Ala-191, Asp-198, Ala-403, Gly-405, Ser-502, Arg-518, Leu-520, and Gln-522 of the chain A of 6m0j as hot.

The prediction are Leu-39, Phe-40, Tyr-41, Gln-42, Ser-43, Ser-44, Leu-45, Ala-46, Ser-47, Trp-48, Asn-49, Tyr-50, Asn-51, Thr-52, Asn-53, Ile-54, Thr-55, Asn-58, Val-59, Gln-60, Asn-61, Met-62, Asn-63, Asn-64, Ala-65, Gly-66, Asp-67, Lys-68, Trp-69, Ser-70, Ala-71, Phe-72, Leu-73, Gly-104, Ser-105, Val-107, Leu-108, Ile-119, Leu-120, Asn-121, Thr-122, Met-123, Ser-124, Thr-125, Ile-126, Tyr-127, Gly-130, Lys-131, Ser-167, Trp-168, Arg-169, Ser-170, Glu-171, Val-172, Gly-173, Lys-174, Gln-175, Leu-176, Arg-177, Pro-178, Leu-179, Tyr-180, Leu-186, Lys-187, Asn-188, Glu-189, Met-190, Ala-191, Arg-192, Ala-193, Asn-194, His-195, Tyr-196, Glu-197, Asp-198, Tyr-199, Gly-200, Asp-201, Tyr-202, Ala-342, His-373, His-374, Glu-375, Met-376, Gly-377, His-378, Glu-398, Gly-399, Phe-400, His-401, Glu-402, Ala-403, Val-404, Gly-405, Glu-406, Ile-407, Met-408, Ser-409, Phe-464, Lys-465, Tyr-497, Cys-498, Asp-499, Pro-500, Ala-501, Ser-502, Leu-503, Phe-504, His-505, Val-506, Ser-507, Asn-508, Arg-514, Tyr-515, Tyr-516, Thr-517, Arg-518, Thr-519, Leu-520, Tyr-521, Gln-522, Phe-523, Gln-524, Phe-525, Gln-526, Met-579, Asn-580, Val-581, Pro-583, and Leu-584.

The SAGNM algorithm recognized the residues Phe-497 and Pro-507 of the chain E of 6m0j as hot.

The predictions are the residues Ile-402, Arg-403, Gly-404, Ser-438, Asn-439, Ser-443, Gly-447, Asn-448, Gln-493, Ser-494, Tyr-495, Gly-496, Phe-497, Gln-498, Pro-499, Thr-500, Asn-501, Val-503, Gly-504, Tyr-505, Gln-506, Pro-507, Tyr-508, Arg-509, Val-510, and Val-511.

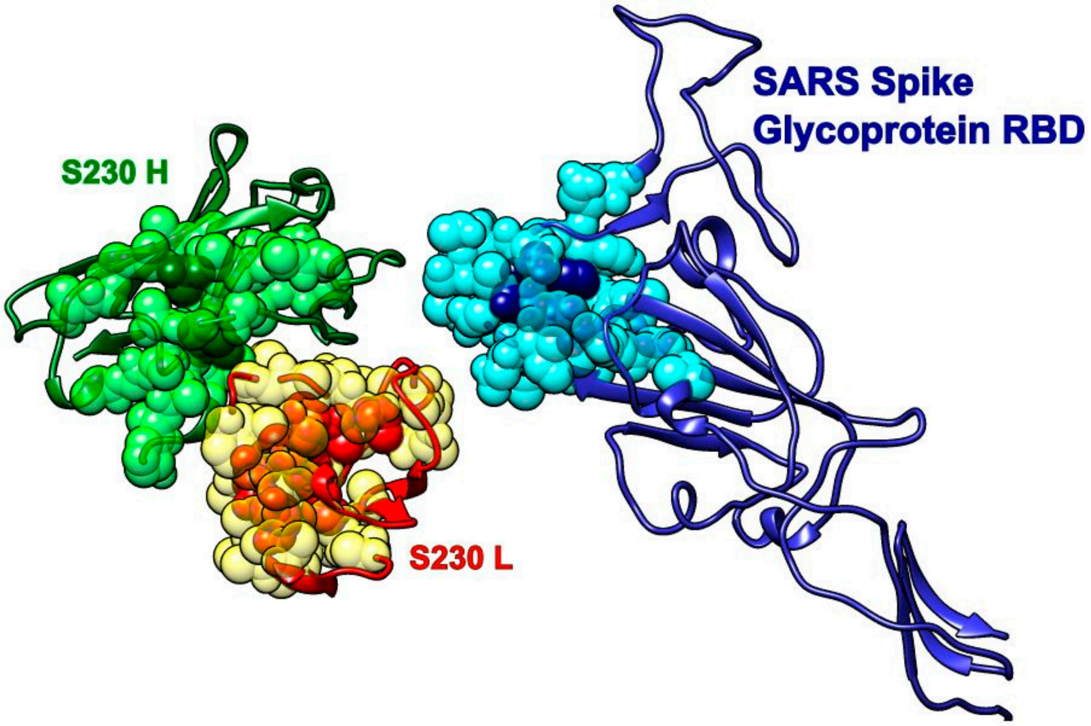


**Figure S10.** Hot residues and predictions for the receptor binding domain (RBD) of SARS-COV spike glycoprotein (Chain A, pdb id 6nb6) with human neutralizing S230 antibody FAB fragment. a) SARS-COV RBD (blue, chain A) with heavy (green, H, and I) and light (red, L, and M) chains. Predictions are cyan (SARS), yellow (S230 light), and light green (S230 heavy).

The SAGNM algorithm recognized the residues Ala-430, Phe-483, and Pro-493 from the RBD domain of SARS-COV spike glycoprotein (Chain A, pdb id 6nb6) as hot.

The predictions are Val-389, Lys-390, Gly-391, Thr-425, Arg-426, Asn-427, Ile-428, Asp-429, Ala-430, Thr-431, Ser-432, Thr-433, Gly-434, Asn-435, Asn-479, Asp-480, Tyr-481, Gly-482, Phe-483, Tyr-484, Thr-485, Thr-486, Thr-487, Ile-489, Gly-490, Tyr-491, Gln-492, Pro-493, Tyr-494, Arg-495, Val-496 and Val-497.

The SAGNM algorithm recognized the residues Glu-6, Val-37, and Tyr-95 from the chain H from 6nb6.pdb as hot. The corresponding predictions are Ala-2, Gln-3, Leu-4, Val-5, Glu-6, Ser-7, Gly-8, Gly-9, Ala-10, Ser-21, Cys-22, Ala-33, Met-34, His-35, Trp-36, Val-37, Arg-38, Gln-39, Ala-40, Pro-41, Gln-46, Trp-47, Leu-48, Thr-91, Ala-92, Val-93, Tyr-94, Tyr-95, Cys-96, Val-97, Thr-98, Gln-99, Gly-118, Gly-120, and Thr-121.

The SAGNM algorithm recognized the residues Glu-6, Val-37, and Tyr-95 from the chain I from 6nb6.pdb as hot. The corresponding predictions are Ala-2, Gln-3, Leu-4, Val-5, Glu-6, Ser-7, Gly-8, Gly-9, Ala-10, Ser-21, Cys-22, Ala-33, Met-34, His-35, Trp-36, Val-37, Arg-38, Gln-39, Ala-40, Pro-41, Gln-46, Trp-47, Leu-48, Thr-91, Ala-92, Val-93, Tyr-94, Tyr-95, Cys-96, Val-97, Thr-98, Gln-99, Gly-118, Gly-120, and Thr-121.

The SAGNM algorithm recognized the residues Gln-6, Cys-23, Trp-40, Phe-41, Ile-53, Tyr-92, Cys-93, Gly-104, Gly-106, and Thr-107 from the chain L from 6nb6.pdb as hot. The corresponding predictions are Val-3, Leu-4, Thr-5, Gln-6, Ser-7, Pro-8, Leu-9, Ser-10, Ala-19, Ser-20, Ile-21, Ser-22, Cys-23, Arg-24, Ser-25, Ser-26, Gln-27, Thr-36, Tyr-37, Leu-38, Asn-39, Trp-40, Phe-41, Gln-42, Gln-43, Arg-44, Pro-45, Pro-49, Arg-50, Arg-51, Leu-52, Ile-53, Tyr-54, Gln-55, Val-56, Ser-57, Asn-58, Arg-59, Phe-76, Val-88, Gly-89, Val-90, Tyr-91, Tyr-92, Cys-93, Met-94, Gln-95, Gly-96, Ser-97, Pro-100, Pro-101, Thr-102, Phe-103, Gly-104, Gln-105, Gly-106, Thr-107, Lys-108, Val-109, Glu-110, and Ile-111.

The SAGNM algorithm recognized the residues Gln-6, Cys-23, Trp-40, Tyr-91, Tyr-92, Thr-107, and Val-109 from the chain M from 6nb6.pdb as hot. The corresponding predictions are Val-3, Leu-4, Thr-5, Gln-6, Ser-7, Pro-8, Leu-9, Ser-10, Leu-11, Pro-12, Ala-19, Ser-20, Ile-21, Ser-22, Cys-23, Arg-

24, Ser-25, Ser-26, Gln-27, Thr-36, Tyr-37, Leu-38, Asn-39, Trp-40, Phe-41, Gln-42, Gln-43, Arg-44, Arg-51, Leu-52, Ile-53, Phe-76, Asp-87, Val-88, Gly-89, Val-90, Tyr-91, Tyr-92, Cys-93, Met-94, Gln-95, Gly-96, Gly-104, Gly-106, Thr-107, Lys-108, Val-109, and Glu-110.

In all cases, the expected number of targets is between 10 and 15%. That corresponds to the first, fastest mode in each case.
